# *In silico* analysis of the fucosylation-associated genome of the human blood fluke *Schistosoma mansoni*: cloning and characterization of the enzymes involved in GDP-L-fucose synthesis and Golgi import

**DOI:** 10.1186/1756-3305-6-201

**Published:** 2013-07-09

**Authors:** Nathan A Peterson, Tavis K Anderson, Xiao-Jun Wu, Timothy P Yoshino

**Affiliations:** 1 Current address: Department of Entomology, College of Agricultural and Life Sciences, University of Wisconsin, 1630 Linden Drive, Madison, WI 53706, USA; 2 Current address: Virus and Prion Research Unit, National Animal Disease Center, USDA, Agricultural Research Service, 1920 Dayton Ave, Ames, IA 50010, USA; 3 Current address: Department of Pathobiological Sciences, School of Veterinary Medicine, University of Wisconsin, 2115 Observatory Drive, Madison, WI 53706, USA

**Keywords:** *Schistosoma mansoni*, Schistosome, Fucose, Fucosylation, GDP-L-fucose synthesis, GDP-L-fucose transport, Fucosyltransferase, Miracidium, Sporocyst

## Abstract

**Background:**

Carbohydrate structures of surface-expressed and secreted/excreted glycoconjugates of the human blood fluke *Schistosoma mansoni* are key determinants that mediate host-parasite interactions in both snail and mammalian hosts. Fucose is a major constituent of these immunologically important glycans, and recent studies have sought to characterize fucosylation-associated enzymes, including the Golgi-localized fucosyltransferases that catalyze the transfer of L-fucose from a GDP-L-fucose donor to an oligosaccharide acceptor. Importantly, GDP-L-fucose is the only nucleotide-sugar donor used by fucosyltransferases and its availability represents a bottleneck in fucosyl-glycotope expression.

**Methods:**

A homology-based genome-wide bioinformatics approach was used to identify and molecularly characterize the enzymes that contribute to GDP-L-fucose synthesis and Golgi import in *S. mansoni*. Putative functions were further investigated through molecular phylogenetic and immunocytochemical analyses.

**Results:**

We identified homologs of GDP-D-mannose-4,6-dehydratase (GMD) and GDP-4-keto-6-deoxy-D-mannose-3,5-epimerase-4-reductase (GMER), which constitute a *de novo* pathway for GDP-L-fucose synthesis, in addition to a GDP-L-fucose transporter (GFT) that putatively imports cytosolic GDP-L-fucose into the Golgi. *In silico* primary sequence analyses identified characteristic Rossman loop and short-chain dehydrogenase/reductase motifs in GMD and GMER as well as 10 transmembrane domains in GFT. All genes are alternatively spliced, generating variants of unknown function. Observed quantitative differences in steady-state transcript levels between miracidia and primary sporocysts may contribute to differential glycotope expression in early larval development. Additionally, analyses of protein expression suggest the occurrence of cytosolic GMD and GMER in the ciliated epidermal plates and tegument of miracidia and primary sporocysts, respectively, which is consistent with previous localization of highly fucosylated glycotopes.

**Conclusions:**

This study is the first to identify and characterize three key genes that are putatively involved in the synthesis and Golgi import of GDP-L-fucose in *S. mansoni* and provides fundamental information regarding their genomic organization, genetic variation, molecular phylogenetics, and developmental expression in intramolluscan larval stages.

## Background

The deoxyhexose sugar L-fucose is a major constituent of an array of immunologically important carbohydrates that are presented on surface-expressed and secreted/excreted glycoconjugates of the human blood fluke *Schistosoma mansoni* (reviewed by [1]). Although the schistosome glycome is perhaps the most extensively characterized among invertebrates, relatively little is known about the enzymatic machinery responsible for its expression. Recent studies by Fitzpatrick *et al*. [2] and Peterson *et al.*[3] inventoried the schistosome α3- and α6-fucosyltransferases (FucTs), which transfer L-fucose from a GDP-L-fucose nucleotide-sugar donor to an oligosaccharide acceptor to create α3 and α6 linkages, respectively. These studies also demonstrated stage- and gender-specific variations in FucT gene transcription, which may contribute to differential fucosyl-glycotope expression that has been reported among stages of *S. mansoni*[4-7].

While the population composition and cellular organization of the expressed glycosyltransferases are key determinants affecting carbohydrate structural diversity, other factors are also important, including nucleotide-sugar donor availability, Golgi membrane dynamics, intralumenal pH, and competition for donor/acceptor substrates [8]. In *S. mansoni*, this means that GDP-L-fucose synthesis and Golgi import, which dictate fucose donor availability in the Golgi, likely contribute to differential fucosyl-glycotope expression. However, to date, no studies have examined these aspects of fucosylation in schistosomes.

**Figure 1:**
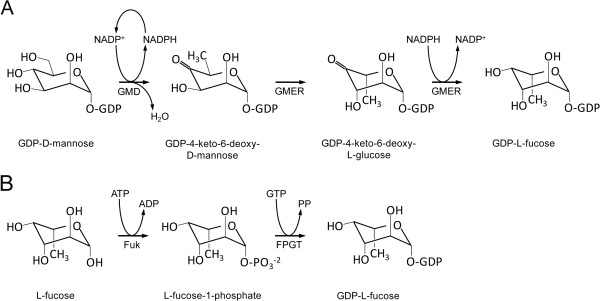
**Schematic diagram of GDP-L-fucose synthesis.** GDP-L-fucose synthesis occurs by two cytosolic pathways, namely the *de novo* and salvage pathways. In *de novo* synthesis **(A)**, GMD with coenzyme NADP^+^ removes one H_2_O-equivalent from GDP-D-mannose to form GDP-4-keto-6-deoxy-D-mannose. Then, GMER catalyzes epimerizations at C3 and C5 followed by an NADPH-dependent reduction of C4 to yield GDP-L-fucose. In the salvage pathway **(B)**, Fuk transfers a single phosphate from ATP to free cytosolic L-fucose, yielding L-fucose-1-phosphate and the byproduct ADP. Next, FPGT transfers GMP from GTP to L-fucose-1-phosphate, producing GDP-L-fucose and pyrophosphate. Evidence presented here strongly supports the exclusive use of the *de novo* synthetic pathway in *S. mansoni*. GMD, GDP-D-mannose-4,6-dehydratase; GMER, GDP-4-keto-6-deoxy-D-mannose-3,5-epimerase-4-reductase; Fuk, L-fucokinase; FPGT, L-fucose-1-phosphate guanylyltransferase.

In general, GDP-L-fucose synthesis is localized in the cytosol and can occur by two possible metabolic pathways, the *de novo* and salvage pathways (reviewed by [9]), which constitute approximately 90% and 10%, respectively, of total GDP-L-fucose synthesis in mammalian cells [10]. In *de novo* synthesis, GDP-D-mannose is converted to GDP-L-fucose in three steps by GDP-D-mannose-4,6-dehydratase (GMD, EC 4.2.1.47) and the bifunctional enzyme GDP-4-keto-6-deoxy-D-mannose-3,5-epimerase-4-reductase (GMER, EC 1.1.1.271; also called GDP-L-fucose synthase). Alternatively, the salvage pathway generates GDP-L-fucose from free cytosolic L-fucose in two steps, which are generally catalyzed by L-fucokinase (Fuk) and L-fucose-1-phosphate guanylyltransferase (FPGT; also called GDP-L-fucose pyrophosphorylase). Both pathways are summarized in Figure 1. GMD and GMER are well conserved across prokaryotic and eukaryotic taxa in terms of both structure and function [11], but the salvage pathway exhibits some variation. While homologs of Fuk and FPGT have been described in several mammalian species [12-15], the salvage pathway in *Bacteroides* and *Arabidopsis* comprises a single bifunctional enzyme (Fkp in *Bacteroides*; FKGP in *Arabidopsis*) that exhibits both Fuk and FPGT activities [16,17]. Elements of a salvage pathway do not exist in *Drosophila*[18] and only a Fuk homolog has been identified in *C. elegans*[11]. How GDP-L-fucose is synthesized in *S. mansoni* is unknown.

In eukaryotes, fucosylation occurs primarily in the Golgi. Consequently, following GDP-L-fucose synthesis in the cytosol, the activated fucose is imported into the Golgi lumen where it can be utilized by Golgi-localized FucTs. This translocation is driven by a GDP-L-fucose transporter (GFT), which couples GDP-L-fucose entry with equimolar exit (i.e., antiportation) of GMP, a downstream byproduct of fucosylation (reviewed by [19]).

Previous studies indicate that GDP-L-fucose synthesis and transport are essential processes in the production of fucosylated glycans. For example, increased expression of GMD, GMER and GFT was linked to higher levels of fucosylation in human hepatocellular carcinoma [20,21] and elevated expression of sialyl Lewis X during inflammation and tumorigenesis [22]. Additionally, Omasa *et al.*[23] observed decreased fucosylation of recombinant human antithrombin III following RNAi-mediated knockdown of GFT in transfected Chinese hamster ovary cells. The essential role of GFT in proper fucosylation is further evidenced in humans by the rare autosomal recessive syndrome leukocyte adhesion deficiency type II (LADII), which is characterized by severe psychomotor and growth retardation, facial malformation, and persistent and recurrent infections with marked neutrophilia [24]. Red blood cells of LADII patients feature a non-fucosylated variant of the H antigen (called the “Bombay” phenotype), and leukocytes lack the fucosylated Lewis-type blood groups that are requisite for extravasation during immune challenge [25]. Importantly, LADII results from a deficiency in GDP-L-fucose transport, which is attributable to mutations in the GFT gene [26-30]. These observations suggest the possibility that GDP-L-fucose synthesis and Golgi import play key roles in the regulated expression of fucosylated glycotopes in *S. mansoni* as well.

In the present study, we used a homology-based genome-wide bioinformatics approach to identify and characterize putative GDP-L-fucose synthesis- and transport-associated genes in *S. mansoni*. This study provides fundamental information about the genomic organization, splicing and molecular phylogenetics of these fucosylation-associated genes as well as important insights regarding their putative roles in glycotope expression in snail-associated larvae, particularly miracidia and primary sporocysts.

## Methods

### Isolation and cultivation of S. mansoni larvae

Ethics statement: Research protocols involving mice, including routine maintenance and care, have been reviewed and approved by the Institutional Animal Care and Use Committee (IACUC) at the University of Wisconsin-Madison under assurance number A3368-01. Generation of antibodies against recombinant proteins was performed by GeneTel Laboratories LLC (Madison, WI, USA) in accordance with protocols reviewed and approved by the Office of Laboratory Animal Welfare (OLAW) at the National Institutes of Health under assurance number A4489-01.

Adult and larval *S. mansoni* (NMRI strain) were collected and cultivated as described by Yoshino and Laursen [31]. Briefly, adults were harvested from infected mice by perforation of the hepatic portal veins, and viable eggs were isolated from liver tissue by homogenization and washed in sterile 0.9% NaCl. Eggs were hatched in artificial pond water [32], and the free-swimming miracidia were either used immediately or transformed to primary sporocysts by cultivation at 26°C in Chernin’s Balanced Salt Solution (CBSS; [33]) containing glucose and trehalose (1 g/L each) as well as penicillin and streptomycin (CBSS^+^). Transformation of most miracidia was complete within 24 h of culture origination. In this study, primary sporocysts were maintained in CBSS^+^ for up to 10 days, with refreshment of the culture medium at 2 and 7 days.

### GDP-L-fucose synthesis and transport gene identification

The amino acid sequences of previously characterized GDP-L-fucose synthesis- and transport-associated genes, including GMDs, GMERs, GFTs, Fuks, FPGTs, Fkp and FKGP, of *Homo sapiens*, *Mus musculus*, *Drosophila melanogaster*, *Caenorhabditis elegans*, *Arabidopsis thaliana*, and *Bacteroides fragilis* were downloaded from Reference Sequence (RefSeq) and GenBank online databases at the National Center for Biotechnology Information (NCBI; accession numbers in Tables 1 and 2) and used as queries in a genome-wide tBLASTn [34] screen of genomic scaffolds and predicted genes to identify homologs in the *Schistosoma mansoni* Database (SchistoDB; [35]).

**Table 1:** NCBI accession numbers (number.version) of GDP-L-fucose synthesis-associated genes referenced in this study

**Source organism**	**Gene identifier **^**a**^	**Nt accession**	**Prot. accession**	**Function **^**b**^	**References**
*Homo sapiens*	**GMDS**	NM_001500.2	NP_001491.1	GMD	[36-39]
	**TSTA3/FX**	NM_003313.3	NP_003304.1	GMER	[20,37,40,41]
	**FUK**	NM_145059.2	NP_659496.2	FUK	[14]
	**FPGT/GFPP**	NM_003838.3	NP_003829.2	FPGT	[13,42,43]
*Mus musculus*	Gmds	NM_146041.2	NP_666153.1	GMD	[22]
	Tsta3	NM_031201.1	NP_112478.1	GMER	[22,44,45]
	**Fuk**	NM_172283.2	NP_758487.2	FUK	[15]
	**Fpgt**	NM_029330.2	NP_083606.2	FPGT	" "
*Danio rerio*	gmds	NM_200489.2	NP_956783.2	GMD	[46,47]
*Drosophila melanogaster*	**Gmd**	NM_135044.3	NP_608888.2	GMD	[11,18]
	**Gmer**	NM_137890.2	NP_611734.1	GMER	" "
*Caenorhabditis elegans*	**gmd-1**	AM231683.1	CAJ77752.1	GMD	[11]
	**gmd-2**	NM_060705.1	NP_493106.1	" "	" "
	**ger-1**	NM_066139.3	NP_498540.1	GMER	" "
*Schistosoma mansoni*	GMD	GU574757.1	ADO17520.1	GMD	Present study
	GMER	GU574758.1	ADO17521.1	GMER	" "
*Mortierella alpina*	GMD	GU299800.1	ADC54120.1	GMD	[48]
	GMER	GU299801.1	ADC54121.1	GMER	" "
*Arabidopsis thaliana*	**GMD1**	NM_126026.3	NP_201429.1	GMD	[11,49]
	**MUR1/GMD2**	NM_114976.3	NP_190685.2	" "	[11,49,50]
	**GER1**	NM_105984.3	NP_177468.2	GMER	[11,51,52]
	**GER2**	NM_101652.2	NP_564040.1	" "	[11]
	**FKGP**	NM_100004.3	NP_563620.1	FUK/FPGT (dual)	[17]
*Bacteroides fragilis*	**Gmd**	CR626927.1	CAH07586.1	GMD	[16]
	**Fcl**	CR626927.1	CAH07585.1	GMER	" "
	**Fkp**	NC_003228.3	YP_212230.1	FUK/FPGT (dual)	[16,53]

^a^ Official gene names/identifiers are provided. Genes in boldface type were used as query sequences to search for homologs in the SchistoDB [35].

^b^ GMD, GDP-D-mannose-4,6-dehydratase; GMER, GDP-4-keto-6-deoxy-D-mannose-3,5-epimerase-4-reductase; FUK, L-fucose kinase; FPGT, fucose-1-phosphate guanylyltransferase; FUK/FPGT, bifunctional L-fucose kinase/fucose-1-phosphate guanylyltransferase.

**Table 2:** NCBI accession numbers (number.version) of nucleotide-sugar transporter genes referenced in this study

**Source organism (tree prefix) **^**a**^	**Gene identifier **^**b**^	**Nt accession**	**Prot. accession**	**NST Substrate(s) **^**c**^	**References**
*Homo sapiens* (Hs)	**SLC35C1**	NM_018389.4	NP_060859.4	GDP-L-Fuc	[21,27,28,54]
	SLC35B4	NM_032826.4	NP_116215.1	UDP-Xyl, UDP-GlcNAc	[55]
	SLC35A3/hUGlcNAcT	NM_012243.1	NP_036375.1	UDP-GlcNAc	[56]
	SLC35D2/hUGTrel8/HFRC1	NM_007001.2	NP_008932.2	UDP-Glc, UDP-GlcNAc, GDP-Man	[57]
	hUGTrel7	AB044343.1	BAB18586.1	UDP-GlcA, UDP-GalNAc	[58]
	hUGT1	D84454.1	BAA12673.1	UDP-Gal, UDP-GalNAc	[59-62]
*Canis lupus* (Cl)	SLC35A3	NM_001003385.1	NP_001003385.1	UDP-GlcNAc	[63]
	SLC35A2	NM_001003059.2	NP_001003059.2	UDP-Gal	[64]
*Mus musculus* (Mm)	**Slc35c1**	NM_211358.2	NP_997597.1	GDP-L-Fuc	[30,65]
	Slc35b4	NM_021435.3	NP_067410.1	UDP-Xyl, UDP-GlcNAc	[66]
	mUGT1	AB027147.1	BAA86885.1	UDP-Gal	[67]
	Slc35a1	NM_011895.3	NP_036025.2	CMP-Sia	[68]
*Cricetulus griseus* (Cgr)	Slc35a1	NM_001246755.1	NM_001246755.1	CMP-Sia	[69]
*Drosophila melanogaster* (Dm)	**Gfr**	NM_141525.1	NP_649782.1	GDP-L-Fuc	[70,71]
	**Efr**	NM_132071.1	NP_572299.1	" "	[72]
	Frc	AB062677.1	BAB62105.1	UDP-GlcA, UDP-GalNAc, UDP-Gal, UDP-GlcNAc, UDP-Xyl	[73,74]
	ugt	AB055493.1	BAB62747.1	UDP-Gal, UDP-GalNAc	[62,75]
*Caenorhabditis elegans* (Ce)	**C50F4.14**	AF323969.1	AAK50396.1	GDP-L-Fuc	[28]
	SQV-7	NM_063035.4	NP_495436.1	UDP-GlcA, UDP-GalNAc, UDP-Gal	[76]
*Schistosoma mansoni* (Sm)	GFT	GU574756.1	ADO17519.1	GDP-L-Fuc (putative)	Present study
*Leishmania donovani* (Ld)	LPG2	U26175.1	AAC46914.1	GDP-Man, GDP-Ara, GDP-Fuc	[77]
*Cryptococcus neoformans* (Cn)	GMT1	XM_571496.1	XP_571496.1	GDP-Man	[78]
	GMT2	XM_571874.1	XP_571874.1	" "	" "
*Saccharomyces cerevisiae* (Sc)	YEA4	NM_001178819.1	NP_010912.1	UDP-GlcNAc	[79]
*Candida albicans* (Ca)	VRG4	AF164627.1	AAK74075.1	GDP-Man	[80]
*Candida glabrata* (Cgl)	Vrg4	AF360395.1	AAK51897.1	GDP-Man	[81]
*Arabidopsis thaliana* (At)	GONST1	AJ314836.1	CAC69066.1	GDP-Man	[82,83]
	GONST2	NM_100603.5	NP_172209.4	" "	[83]
	AtUTr1	AY115566.1	AAM48281.1	UDP-Gal, UDP-Glc	[84]
	NST-K1	NM_179196.1	NP_849527.1	UDP-Gal	[85]
	udpgalt1	AJ633720.1	CAG18176.1	" "	[86]
	udpgalt2	AJ633721.1	CAG18177.1	" "	" "

^a^ “tree prefix” refers to nomenclature applied in phylogenetic analyses of NSTs (Figure 6, in Additional file 3: Figure S2).

^b^ Official gene names/identifiers are provided. Genes in boldface type were used as query sequences to search for GDP-L-fucose transporter homologs in the SchistoDB [35].

^c^ NST activity has been demonstrated for these substrates. GDP-Fuc, GDP-L-fucose; UDP-Xyl, UDP-D-xylose; UDP-GlcNAc, UDP-D-N-acetylglucosamine; UDP-Glc, UDP-D-glucose; UDP-GlcA, UDP-D-glucuronic acid; UDP-GalNAc, UDP-D-N-acetylgalactosamine; UDP-Gal, UDP-D-galactose; GDP-Man, GDP-D-mannose; CMP-Sia, CMP-sialic acid; GDP-Ara, GDP-D-arabinose.

### Primer design

The oligonucleotide primers used in this study were designed using Vector NTI Advance 11.0 software (Invitrogen, Eugene, OR, USA) and the IDT SciTools suite [87] based on available SchistoDB-derived genomic sequence information as well as data obtained by this study, and custom DNA oligonucleotides were purchased from Integrated DNA Technologies (IDT, Coralville, IA, USA). A complete list of primer sequences used in this study is provided in (Additional file 1: Table S1A-E).

### Reverse transcriptase-PCR and rapid amplification of cDNA ends for GMD, GMER, and GFT transcript sequencing

Kits and reagents for molecular assays were used according to the manufacturers’ recommendations unless otherwise indicated. Primers used for reverse transcription (RT)-PCR and rapid amplification of cDNA ends (RACE) are provided (see Additional file 1: Table S1A-C). RT-PCR and RACE protocols were performed as detailed in [3] and are summarized as follows: Miracidia, 2-day *in vitro*-cultivated primary sporocysts and mixed-sex adults (i.e., pooled male and female worms) were washed with artificial pond water (miracidia), CBSS (sporocysts) or mammalian phosphate-buffered saline (8.41 mM Na_2_HPO_4_, 1.65 mM NaH_2_PO_4_·H_2_O, 146.4 mM NaCl, pH 7.4; adults), and total (“raw”) RNA was extracted using TRIzol® Reagent (Invitrogen). Genomic contamination was removed with TURBO™ DNase (Applied Biosystems, Foster City, CA, USA), and the resultant DNA-free RNA was converted to RT-PCR-ready cDNA using the SuperScript® III First-Strand Synthesis System (Invitrogen). Reverse transcriptase-PCR reactions were prepared with GoTaq® PCR reagents (Promega, Madison, WI, USA), and amplification products were QIAquick-purified (Qiagen, Germantown, MD, USA), ligated into pCR®4-TOPO® sequencing vector (Invitrogen) and cloned in One Shot® TOP10 Chemically Competent *Escherichia coli* (Invitrogen). Inserts in QIAprep-isolated plasmids (Qiagen) were sequenced by BigDye Terminator dideoxy PCR sequencing (Applied Biosystems) and, following purification with Agencourt® CleanSEQ® magnetic beads (Beckman Coulter, Brea, CA, USA), reaction products were read by the DNA Sequence Laboratory at the University of Wisconsin Biotechnology Center (Madison, WI, USA). Following RT-PCR confirmation of gene transcription, RACE-ready cDNA was prepared from TRIzol®-derived DNA-free total parasite RNA using a SMART™/SMARTer™ RACE cDNA Amplification Kit (Clontech, Mountain View, CA, USA), and gene-specific cDNA ends were PCR-amplified using an Advantage® 2 PCR Kit (Clontech). Amplification products were isolated, cloned and sequenced as above. Transcript sequences were assembled from the compiled sequence data and edited using Vector NTI Advance 11.0 software. The complete coding sequences (CDSs) were then verified by RT-PCR amplification and sequencing (as above) using primers flanking the open reading frames (ORFs).

### Phylogenetic analysis of nucleotide-sugar transporters

Representative amino acid sequences of functionally characterized nucleotide-sugar transporters were compiled from RefSeq and GenBank databases with our data from *S. mansoni* (Table 2). Sequences were aligned using default settings in MUSCLE v 3.6 [88], with subsequent manual correction in Mesquite [89]. A guide tree was developed for Bayesian phylogenetic inference using neighbor-joining methods in FastTree v 2.0.1 [90] with a Jukes-Cantor + CAT model. Analyses were then performed using mixed amino acid models within MrBayes v 3.1.2 [91] with two parallel runs of four Markov chain Monte Carlo (MCMC) chains, each for five million generations, with subsampling every 100th generation. To ensure the tree search was not trapped at local optima, two independent replicates were conducted [92]. Stationarity of molecular evolutionary parameters was assessed at effective sample sizes >400 in Tracer v1.5 [93]. Additionally, convergence of the MCMC chains was evaluated using the online program AWTY [94]. Trees prior to stationarity were burned-in, and remaining trees were used to assess posterior probabilities for nodal support.

### Real-time quantitative PCR analysis of GMD, GMER, and GFT mRNA expression in miracidia and primary sporocysts of S. mansoni

Real-time quantitative (q)PCR protocols used in this study were performed according to the recommendations by Applied Biosystems [95], including strict criteria for qPCR primer design, validation and optimization. Relative transcript abundance in miracidia and primary sporocysts was examined using the comparative C_T_ (ΔΔC_T_) method. ATP synthase f (herein termed “*ATPsf*”; SAGE tag 195 corresponding to Smp_140480 in the SchistoDB) and the *GroES* chaperonin (SAGE tag 132 corresponding to Smp_097380) were selected as endogenous calibrators based on SAGE data [96], which indicate stable expression between miracidia and primary sporocysts. The compatibility of calibrator and gene of interest (GOI) qPCR primers under normal reaction conditions was assessed by plotting ΔC_T_ at 10-fold dilutions of cDNA input and determining the slope of the resultant semi-log regression line; primer efficiencies were deemed compatible if the absolute value of the slope was less than 0.1. Validated calibrator and GOI primer sequences are listed in Additional file 1: Table S1D.

Miracidia and *in vitro*-cultivated primary sporocysts were washed with artificial pond water and CBSS, respectively, followed by extraction of total RNA and immediate preparation of first-strand cDNA as above. It should be noted that RNA integrity was not routinely assessed prior to cDNA synthesis (as per MIQE guidelines [97]) due to limited raw RNA yields; however, integrity in select samples was visually inspected via electrophoretic fractionation. Also, raw and DNA-free RNA concentrations were estimated using a NanoDrop 1000 Spectrophotometer (Thermo Fisher Scientific, Waltham, MA, USA), and only samples exhibiting A_260_:A_280_ and A_260_:A_230_ ratios >1.8 were processed for inclusion in qPCR analyses. Real-time qPCR reactions (50 μL/rxn) were performed in triplicate using an ABI 7300 Real-Time PCR System (Applied Biosystems), with reaction mixtures comprising 1× SYBR Green PCR Master Mix (Applied Biosystems), 20 ng RNA input-equivalents of parasite cDNA and gene-specific primers (100 nM each forward and reverse for *GMD*, *GMER* and *GFT*; 200 nM each for *GroES* and *ATPsf*). Cycling parameters included an initial denaturation at 95°C for 10 min followed by 40 cycles of 95°C for 15 sec and 60°C for 1 min. Amplification fidelity was confirmed by post-cycling thermal dissociation and agarose gel fractionation of qPCR products. The geometric mean of *ATPsf* and *GroES* C_T_ values was used to normalize GOI C_T_ values such that ΔC_T_ = C_T-GOI_ - C_T-GeoMean(*ATPsf*, *GroES*)_, and ΔC_T_ values were compared across three independent biological replicates using iterative heteroscedastic two-sample t- and Wilcoxon rank sum tests, with significance set at *p*≤0.05 and *p*=0.10, respectively. It should be noted that the nonparametric Wilcoxon rank sum test lacks statistical power when sample size is low (e.g., n=3) and a *p*-value of 0.10 is acceptable in the current analyses.

### Expression and purification of recombinant GMD and GMER for antibody production

Heterologous expression and purification of recombinant GMD and GMER proteins were performed using the GST fusion vector pGEX-6P-1 (GE Healthcare, Piscataway, NJ, USA), which incorporates N-terminal GST and an interceding PreScission™ Protease cleavage site, according to recommendations by Amersham Biosciences (GE Healthcare). The complete CDSs of *GMD* and *GMER* were amplified from RT-PCR-ready larval cDNA (generated as above) using 5′-tagged primers designed to incorporate BamHI or EcoRI restriction sites at the amplicon ends (*GMD*, BamHI-forward and EcoRI-reverse; *GMER*, BamHI-forward and BamHI-reverse; in Additional file 1: Table S1E). Reverse transcriptase-PCR reactions (25 μL/rxn) comprised 2.5 U GoTaq® Flexi DNA Polymerase, 1× Green GoTaq® Flexi Reaction Buffer, 400 nM each forward and reverse restriction-tagged primers, 1.6 mM dNTP mix (400 μM each), 1.5 mM MgCl_2_ and ~200 ng RNA input-equivalents of larval cDNA. The thermal profile included initial denaturation at 94°C for 3 min, 40 cycles of 94°C for 15 sec, 58°C for 30 sec and 72°C for 2 min, and final extension at 72°C for 10 min. Following electrophoretic fractionation and ethidium bromide-meditated visualization in 1% agarose gel, the tagged amplification products were isolated by QIAquick gel extraction. To prepare *GMD* and *GMER* expression constructs, stock pGEX-6P-1 vector and purified restriction-tagged PCR products were digested with BamHI-HF™ *and* EcoRI-HF™ (New England BioLabs, Ipswich, MA, USA) (double digest, *GMD*) or with BamHI-HF™ alone (*GMER*). Double digests (25 μL/rxn) included 500 U each BamHI-HF™ and EcoRI-HF™, 1× NEBuffer 4 and ~4 μg pGEX-6P-1 stock vector or restriction-tagged *GMD* amplicon. The protocol for single digests of restriction-tagged *GMER* and the stock pGEX-6P-1 vector excluded EcoRI-HF™. In both schemes, reactions were incubated at 37°C for 2 h. To prevent self-ligation, 2 U calf intestinal alkaline phosphatase (CIP, New England BioLabs) in 1× NEBuffer 4 was added to the linearized pGEX-6P-1 vector, and reactions (30 μL total volume) were incubated at 37°C for 1 h. Both CIP-treated pGEX-6P-1 vector and restriction-digested *GMD*/*GMER* amplicon were purified by electrophoretic fractionation in 1% agarose gel, and the DNA fragments were isolated by QIAquick gel extraction. Next, *GMD*/*GMER* amplicon and CIP-treated pGEX-6P-1 vector were combined (5:1 cohesive ends ratio) with 2000 U T4 DNA Ligase and 1× T4 DNA Ligase Reaction Buffer (New England BioLabs), and ligation reactions were incubated at 22°C for 30 min followed by 65°C for 15 min. Recombinant plasmids were cloned in One Shot® BL21 (DE3) Chemically Competent *E. coli* (Invitrogen), and QIAprep-purified plasmids were sequenced using pGEX 5′ Sequencing Primer 5′-d[GGGCTGGCAAGCCACGTTTGGTG]-3 and pGEX 3′ Sequencing Primer 5′-d[CCGGGAGCTGCATGTGTCAGAGG]-3′ (GE Healthcare).

To express GMD and GMER proteins, plasmid-bearing BL21 cells were grown overnight at 37°C in 2YT medium (1.6% tryptone, 1.0% yeast extract, 0.5% NaCl) containing 100 μg/mL ampicillin (2YTA). Overnight cultures were diluted 1:40 with 2.4 L 2YTA (6*400 mL/flask), and cells were grown at 37°C until A_600_ reached ~0.6. Then cultures were induced with 0.1 mM isopropyl β-D-1-thiogalactopyranoside (Sigma-Aldrich, St. Louis, MO, USA) and incubated overnight (~16 h) at 26°C with shaking at 200 rpm. Cells were pelleted by centrifugation for 10 min at 7000 *g* and 4°C, after which pellets were freeze-thawed three times and resuspended in GST-A buffer (20 mM Tris, 1 M NaCl, 0.2 mM EDTA, 1 mM DTT) containing 1× Protease Inhibitor Cocktail Set III (EMD Chemicals, Gibbstown, NJ, USA). Cells were disrupted on ice by four pulses for 25 sec/pulse at output 5 and duty cycle 40% using an S-450A Branson® Sonifier (Branson Ultrasonics Corp., Danbury, CT, USA). Triton X-100 (Sigma-Aldrich) was added to 1.0% final concentration, and homogenates were gently mixed for 30 min at 4°C. After centrifugation for 10 min at 12000 *g* and 4°C to remove cellular debris, supernatants were filtered with a 0.45 μm Nalgene® syringe filter (Thermo Fisher Scientific), and the GST fusion proteins were affinity-purified on 1 mL GSTrap FF columns (GE Healthcare) using a TRIS™ peristaltic pump (Teledyne Isco, Lincoln, NE, USA). Columns were primed with 10 mL phosphate-buffered saline (PBS: 10 mM Na_2_HPO_4_, 1.8 mM KH_2_PO_4_, 140 mM NaCl, 2.7 mM KCl, pH 7.3) at a flow rate of 1.0 mL/min, loaded with filtered extract at 0.5 mL/min, washed with 10 mL PBS at 1.0 mL/min, equilibrated with PreScission™ cleavage buffer (PCB: 50 mM Tris–HCl, 150 mM NaCl, 1 mM EDTA, 1 mM dithiothreitol, pH 7.5) at 1.0 mL/min, incubated 4 h at 4°C with 160 U PreScission™ Protease in 1 mL PCB and eluted from the column with 3 mL PCB. Eluates containing cleaved GMD/GMER were concentrated 10-fold with an Amicon® Ultra-4 30 kDa MWCO centrifugal filter (Millipore, Billerica, MA, USA) and fractionated on a preparative 10% polyacrylamide gel (~1-2 mg protein load). Proteins were visualized with Bio-Safe Coomassie Stain (Bio-Rad Laboratories, Hercules, CA, USA), and GMD and GMER bands were excised and stored at −20°C in PBS. Polyclonal chicken IgY antibodies against gel-isolated GMD and GMER proteins were commercially produced by GeneTel Laboratories LLC (Madison, WI, USA).

### Antibody purification using blotted recombinant GMD and GMER proteins

To reduce nonspecific binding and cross-reactivity of GMD and GMER chicken IgY antibodies, 200 μg purified GMD/GMER protein was fractionated in 12.5% polyacrylamide gel and electroblotted for 1.5 h at 100 mA onto 0.2 μm nitrocellulose (Bio-Rad Laboratories) using a TE 77 Semi-Dry Transfer Unit (Hoefer, San Francisco, CA, USA). Following transfer, the membrane-immobilized proteins were visualized with Ponceau S stain (Sigma-Aldrich), and bands were excised by razorblade. After destaining, the protein-bearing membrane strips were blocked overnight at 4°C with 5% nonfat dry milk in tris-buffered saline (TBS: 20 mM Tris, 150 mM NaCl, pH 7.5), rinsed two times with TBS containing 0.05% Tween® 20 (Thermo Fisher Scientific) (TBST), incubated overnight at 4°C with 10 mL crude pre-immune or gene-specific chicken IgY and washed three times with TBST. Finally, bound antibodies were eluted twice by incubation for 10 min in 5 mL 0.1 M Glycine-HCl (pH 2.7), with eluates being immediately neutralized with 400 μL 2 M tris (pH 8.0) followed by dialysis in PBS overnight at 4°C using a 7K MWCO Pierce Slide-A-Lyzer® Dialysis Cassette (Thermo Fisher Scientific). This antibody isolation procedure was repeated twice more using the same antigen-bound strips. Dialyzed eluates were combined and concentrated ~250-fold with a 9K MWCO Pierce Protein Concentrator (Thermo Fisher Scientific), and stored for later use at 4°C in 50% glycerol.

### Preparation of cytosolic, membrane/organelle, nuclear and cytoskeletal protein fractions from larval S. mansoni

Subcellular fractionation of miracidia, primary sporocysts and mixed-sex adult worms was performed using a modification of the ProteoExtract® Subcellular Proteome Extraction Kit (EMD Chemicals) protocol, which was originally optimized for use with mammalian cell/tissue samples. Parasites were gently washed four times with artificial pond water (miracidia), CBSS (sporocysts) or mammalian PBS (adults), followed by two washes with Calbiochem® Wash Buffer (kit component). After the final wash, the parasites were pelleted by centrifugation for 1 min at 300 *g* and 4°C, resuspended in 1.5 mL Extraction Buffer I containing 1× protease inhibitor cocktail (PIC) (kit components), and gently agitated for 10 min at 4°C on a LABQUAKE® Rotatory shaker (Barnstead/Thermolyne, Dubuque, IA, USA). The parasite residua were pelleted by centrifugation for 10 min at 1100 *g* and 4°C, and the supernatant (cytosolic fraction, F1) was transferred to a clean tube on ice. Residua were then resuspended in 1.5 mL Extraction Buffer II containing 1× PIC (kit components) and incubated 30 min at 4°C on the rotary shaker. Following centrifugation for 10 min at 6500 *g* and 4°C, the supernatant (membrane/organelle fraction, F2) was placed on ice. Parasite residua were resuspended again in 0.75 mL Extraction Buffer III containing 1× PIC and 562.5 U Benzonase® (kit components), and suspensions were incubated on the rotary shaker for 10 min at 4°C. The insoluble material was pelleted by centrifugation for 10 min at 8200 *g* and 4°C, and the supernatant (nuclear fraction, F3) was set aside on ice. Finally, the residua were resuspended in 0.75 mL Extraction Buffer IV containing 1× PIC (kit components) and incubated for 30 min at room temperature on the rotary shaker. Insoluble cell debris was pelleted for the last time by centrifugation at 8200 *g* and room temperature and the final fraction (cytoskeletal fraction, F4) was set on ice. All fractions were then dialyzed in PBS overnight at 4°C using 6-8K MWCO D-Tube™ Dialyzers (EMD Chemicals) and concentrated ~15 fold with a Microcon® YM-10 Centrifugal Filter Device (Millipore).

### SDS-PAGE and western blot analyses of schistosome subcellular protein fractions

Subcellular protein extracts (8.5 μg protein/lane) were fractionated in 12.5% polyacrylamide gel, and proteins were electroblotted for 1.5 h at 100 mA onto 0.2 μm nitrocellulose. Membranes were blocked overnight with 5% milk in TBS at 4°C, incubated 2 h at room temperature with membrane-purified chicken IgY diluted 1/20 with 5% milk in TBS, washed three times with TBST (5 min/wash), treated 2 h with alkaline phosphatase-conjugated rabbit α-chicken IgY (GeneTel Laboratories LLC) diluted 1/10000 with 5% milk in TBST, washed three more times with TBST and developed in alkaline phosphatase buffer (100 mM Tris, 100 mM NaCl, 50 mM MgCl_2_, pH 9.5) containing 5-bromo-4-chloro-3-indoylphosphate *p*-toluidine salt and nitro-blue tetrazolium chloride (Thermo Fisher Scientific).

### Processing of schistosome larvae for confocal laser scanning microscopy

Preparation of parasite larvae for confocal laser scanning microscopy was performed as described by Peterson *et al*. [7] with modifications. All in-tube washes and treatments were performed at 4°C on a rotary shaker, and parasite larvae were pelleted by centrifugation for 2 min at 300 *g* between incubations. Briefly, miracidia and 2- and 10-day *in vitro*-cultivated primary sporocysts were washed five times with artificial pond water (miracidia) or snail PBS (sPBS: 8.41 mM Na_2_HPO_4_, 1.65 mM NaH_2_PO_4_·H_2_O, 45.34 mM NaCl, pH 7.4; sporocysts) and transferred to a Sigmacote®-treated (Sigma-Aldrich) microfuge tube. Larvae were simultaneously fixed and permeabilized by overnight incubation in 4% paraformaldehyde and 1% Triton X-100 (Sigma-Aldrich) in sPBS (pH 7.2), washed five times with 2% bovine serum albumin (BSA) and 0.02% azide in sPBS (15 min/wash) and blocked overnight in sPBS containing 5% BSA and 0.02% azide (blocking buffer). Blocked larvae were incubated for 3 days in membrane-purified anti-GMD/GMER antibody concentrates diluted 1/100 in blocking buffer containing 0.1% Tween® 20. Following primary treatment, larvae were washed six times with 1% BSA, 0.02% azide and 0.1% Tween® 20 in sPBS (wash buffer) (20 min/wash) and treated overnight with a mixture of Hoechst 33258 dye (50 μg/mL; Invitrogen), Alexa Fluor®546-conjugated phalloidin (7.5 U/mL; Invitrogen) and Alexa Fluor®488-conjugated goat anti-chicken IgY secondary antibody (4 μg/mL, Invitrogen) in blocking buffer containing 0.1% Tween® 20. Finally, larvae were washed six times with wash buffer (five 20 min washes, one overnight wash), mounted in Vectashield® mounting medium (Vector Laboratories, Burlingame, CA, USA) and imaged at 600× total magnification under oil immersion using an A1R confocal microscope (Nikon Instruments Inc., Melville, NY, USA) equipped with laser lines of 408 nm, 488 nm and 561 nm for the excitation of Hoechst, Alexa Fluor®488 and Alexa Fluor®546 dyes, respectively. Confocal fluorescence images were processed using Adobe Photoshop CS v9.0 (Adobe Systems Inc., San Jose, CA, USA), and antibody reactivities were assessed against secondary-only and membrane-purified preimmune controls.

## Results and discussion

### Composition, genomic organization, and splicing of schistosome GDP-L-fucose synthesis and transport genes

An exhaustive homology-based search of the *Schistosoma mansoni* Database (SchistoDB; [35]) using a diversity of previously characterized GDP-L-fucose synthesis- and transport-associated enzymes (see Tables 1–2) identified three homologs in the schistosome genome, herein termed *GMD*, *GMER* and *GFT* (genes and corresponding SchistoDB annotations listed in Table 3). *GMD* and *GMER* putatively constitute a complete *de novo* pathway for GDP-L-fucose synthesis. No homologs of salvage pathway-associated genes (*Fuk*, *FPGT*, *Fkp*, *FKGP*) were identified, suggesting that GDP-L-fucose synthesis in *S. mansoni* occurs only by *de novo* conversion of GDP-D-mannose. Unlike *Caenorhabditis* and *Arabidopsis*, which encode multiple paralogs of GMD and GMER [11,49], only one homolog of each gene occurs in *S. mansoni*. In addition to known Golgi-associated GFTs, search queries included the ER-resident transporter *Efr*, which imports GDP-L-fucose donor substrates for consumption by ER-associated protein O-FucTs in *Drosophila*. These searches failed to identify a homologous ER-type GFT in *S. mansoni* despite the previous finding that schistosomes express two putative ER-resident protein O-fucosyltransferases [3]. Notably, Ishikawa *et al*. [72] observed that *Drosophila* Golgi- and ER-resident GFTs (Gfr and Efr, respectively) function redundantly in the O-fucosylation of Notch receptor, suggesting the existence of two pathways for supplying GDP-L-fucose to ER-resident protein O-FucTs. Therefore, a second, ER-type GFT may not be necessary for O-fucosylation in *S. mansoni*.

**Table 3:** **Genomic organization of GDP-D-mannose-4,6-dehydratase (*GMD*), GDP-4-keto-6-deoxy-D-mannose-3,5-epimerase-4-reductase (*GMER*) and a GDP-L-fucose transporter (*GFT*) in *Schistosoma mansoni***

**Gene**	**Gene ID **^**a**^	**Scaffold ID **^**a**^	**Approx. size (bp)**	**No. of exons**	**ORF length (nt) **^**b**^	**Prot. length (aa) **^**b**^
*GMD*	Smp_153490	Smp_scaff000159	>4,911	10	1,089	363
*GMER*	Smp_104720	Smp_scaff001995	≥7,696	7	954	318
*GFT*	Smp_155830	Smp_scaff000188	>13,167	11	1,149	383

^a^ Smp gene and scaffold IDs refer to nomenclature in the SchistoDB [35].

^b^ ORF and protein sizes are provided for the main/major transcripts. Alternative splicing may alter ORF length and protein coding.

**Figure 2:**
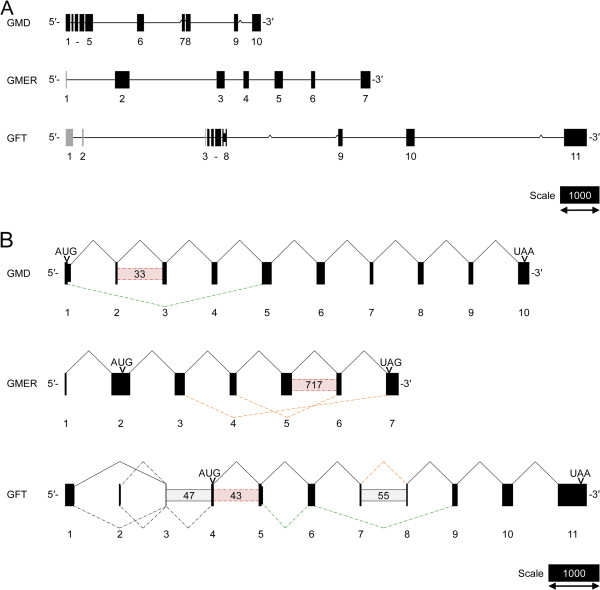
**Genomic organization and splicing of GDP-L-fucose synthesis- and transport-associated genes in *Schistosoma mansoni*.** The mRNA transcript sequences of *GMD*, *GMER* and *GFT* were mapped onto genomic scaffolds in the SchistoDB [35]**(A)**. Exons (boxes, numbered below) and introns (connecting lines) are drawn to scale (bar = 1000 nt) with *GMD*/*GMER*/*GFT*-coding elements, including exons and a subset of retained introns, depicted as black boxes and non-coding exons depicted as gray boxes. Caret marks indicate gaps in the genomic sequence. Alternative splicing, including exon skipping, intron retention, mutual exclusivity and use of alternate splice donor sites, was observed during transcript sequencing **(B)**. Bent connectors indicate splicing between exons (boxes, numbered below), with solid lines representing splicing in the main/major full-length *GMD*/*GMER*/*GFT*-coding transcripts and dotted lines representing alternative splice events. In this panel, exons are drawn to scale (bar = 1000 nt) and spacing of exons is arbitrary. Interexonic boxes represent retained introns (estimated lengths in parentheses) with solid outlines signifying retention in the main/major transcript and dotted lines indicating retention in other isoforms. The positions of the prototypical start and stop codons (AUG and UAA/UAG, respectively) are shown. Colors convey the *in silico* consequences of splicing: black, conservation of the prototypical ORF; red, introduction of a PTC; orange, induction of a downstream frameshift; green, in-frame deletion/addition.

To confirm mRNA expression of *GMD*, *GMER* and *GFT* in *S. mansoni* and obtain full-length CDSs, transcript sequences were RT-PCR and RACE-amplified from miracidial, primary sporocyst and adult worm cDNAs. Complete nucleotide sequences were submitted to GenBank at NCBI (accession numbers in Tables 1–2). While *GMD* and *GMER* sequence data generally validate the corresponding SchistoDB predictions, the data indicate that annotation Smp_155830 erroneously combines a portion of the *GFT* CDS with an upsteam gag-pol polyprotein-coding gene, which comprises ~65% of the predicted *GFT* CDS. Mapping sequence data onto the corresponding SchistoDB-derived genomic scaffolds demonstrated that schistosome *GMD*, *GMER* and *GFT* are all multiexonic, with CDSs spanning 10, 6 and 8 exons, respectively (Table 3, Figure 2A).

Alternative splicing was observed for all schistosome GDP-L-fucose synthesis- and transport-associated genes (Figure 2B). Because many of these observations were based on data obtained by RT-PCR and RACE, which target specific sections of each transcript rather than complete CDSs, the relationships among alternative splice events (i.e., whether splice events occur co-dependently in the formation of particular isoforms) are largely unknown. Most modes of alternative splicing were observed, including exon skipping (*GMD*, *GMER* and *GFT*), intron retention (*GMD*, *GMER* and *GFT*), mutual exclusion (e.g., exons 1 and 2 of *GFT*) and use of alternative splice donor sites (*GMD* and *GMER*). An *in silico* analysis to determine the consequences of alternative splicing revealed that many of these events altered protein coding by introducing a premature termination codon (PTC), forcing a downstream frameshift, or effecting an in-frame deletion or addition. However, additional studies are required to determine the true biochemical effects of these variations.

In eukaryotes, alternative splicing is often an important source of phenotypic complexity, which is driven by splice-mediated expansion of the proteome, posttranscriptional gene regulation (e.g., introduction of a PTC that leads to nonsense-mediated decay) and alteration of cis-regulatory elements that control mRNA translation efficiency, stability and localization (reviewed by [98]). Additionally, in many biological systems, alternative splicing is an important mechanism of modulating physiological activity during development, differentiation and stress responses, and such developmentally regulated alternative splicing has been well documented in *S. mansoni* (e.g., [99-101]). While a comprehensive investigation of splice variation in the context of parasite development was beyond the scope of the present study, the data feature multiple examples of variant splice events that potentially modulate *GMD*, *GMER* and *GFT* expression. For instance, the observed splice-mediated introduction of PTCs and frameshifts could target the affected *GMD*, *GMER* and *GFT* transcripts for nonsense-mediated decay, and developmental regulation of these processes could yield stage- and/or tissue-specific GDP-L-fucose synthesis and transport activities. Moreover, this could affect FucT activity in the Golgi and ultimately determine the developmental expression of fucosylated glycotopes.

### In silico characterization of schistosome GMD, GMER, and GFT

**Figure 3:**
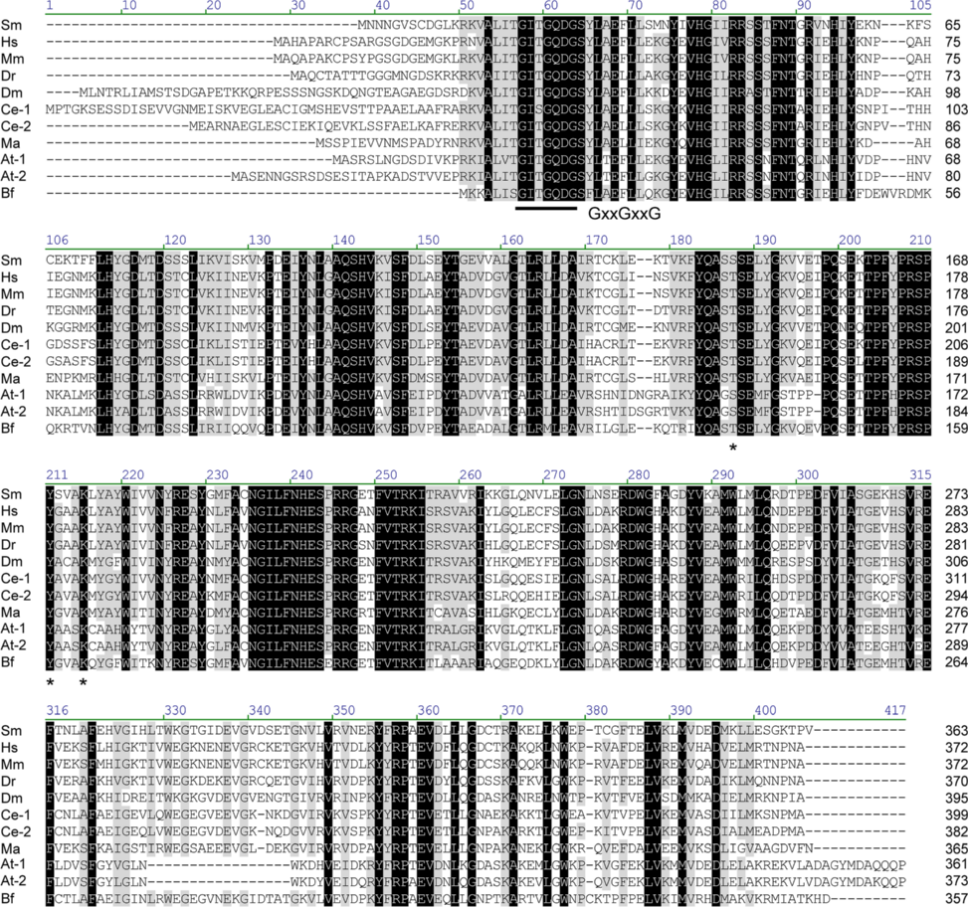
**Amino acid alignment of GDP-D-mannose-4,6-dehydratases.** The predicted amino acid sequence of schistosome GMD (Sm) is compared to GMDs of humans (Hs), *Mus musculus* (Mm), *Danio rerio* (Dr), *Drosophila melanogaster* (Dm), *Caenorhabditis elegans* (Ce-1, Ce-2), *Mortierella alpina* (Ma), *Arabidopsis thaliana* (At-1, At-2) and *Bacteroides fragilis* (Bf) (accession numbers in Table 1). Alignment position is indicated above each block, and sequence length is reported to the right of each line. Positions exhibiting greater than 80% conservation are highlighted in gray, and identities are identified in black. A well-conserved glycine-rich phosphate-binding loop (GxxGxxG), which is key to water-mediated hydrogen bonding between the Rossman folds of redox-associated enzymes and the pyrophosphates of dinucleotide enzyme cofactors (e.g., NAD^+^/NADP^+^) [102], is underlined. Also, the catalytically important [S/T]-Y-K triad common to members of the SDR family of enzymes is indicated by asterisks [103,104]. Vector NTI Advance 11.0 software alignment settings: BLOSUM45 matrix, gap opening penalty = 12, gap extension penalty = 0.1, gap separation penalty range = 0, no residue-specific or hydrophobic residue gaps.

**Figure 4:**
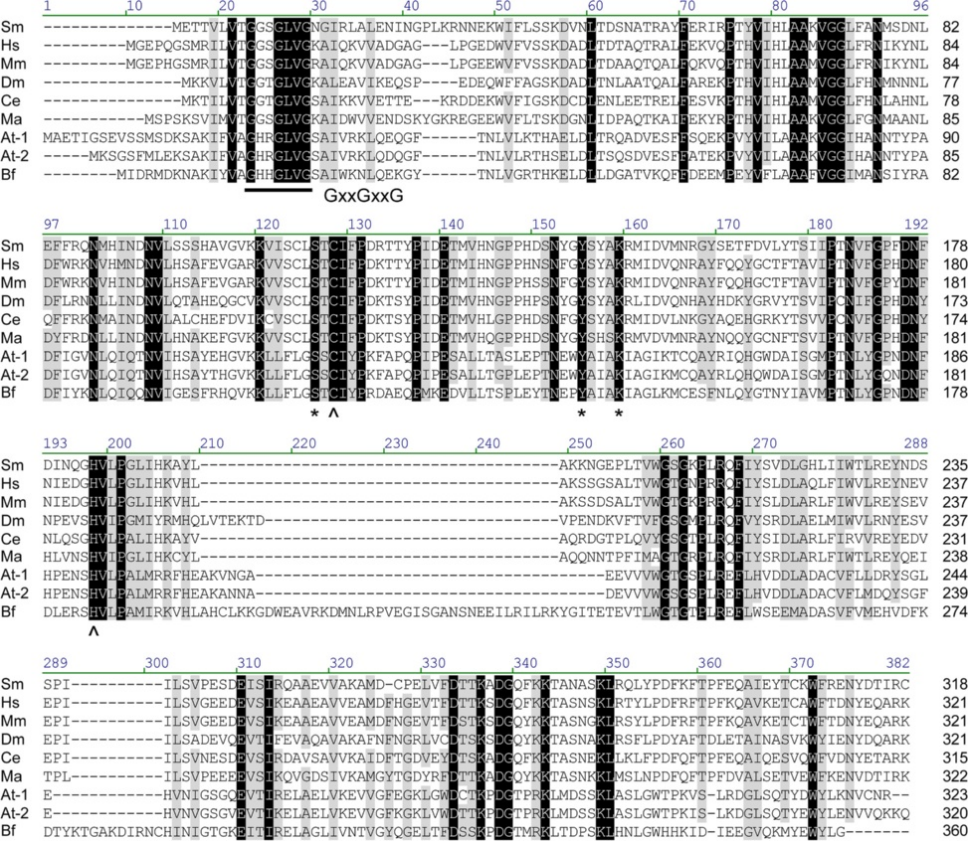
**Amino acid alignment of GDP-4-keto-6-deoxy-D-mannose-3,5-epimerase-4-reductases.** The predicted amino acid sequence of schistosome GMER (Sm) is compared to GMERs of humans (Hs), *Mus musculus* (Mm), *Drosophila melanogaster* (Dm), *Caenorhabditis elegans* (Ce), *Mortierella alpina* (Ma), *Arabidopsis thaliana* (At-1, At-2) and *Bacteroides fragilis* (Bf) (accession numbers in Table 1). Alignment position is indicated above each block, and sequence length is reported to the right of each line. Positions of identity are indicated in black, and gray-highlighted positions are greater than 80% conserved among the sampled sequences. A well-conserved glycine-rich phosphate-binding loop (GxxGxxG), which mediates the binding of dinucleotide cofactors (e.g., NAD^+^/NADP^+^) by redox-associated enzymes [102], is underlined. Additionally, the catalytically important [S/T]-Y-K triad of the short-chain dehydrogenase/reductase-type enzymes is indicated by asterisks (*), and residues thought to be involved in proton exchange between GMER and its epimerization reaction intermediates are marked with carets (^) [105]. Vector NTI Advance 11.0 software alignment settings: BLOSUM45 matrix, gap opening penalty = 12, gap extension penalty = 0.1, gap separation penalty range = 0, no residue-specific or hydrophobic residue gaps.

To provide support for their putative roles in GDP-L-fucose synthesis and transport, the predicted amino acid sequences of schistosome GMD, GMER and GFT were compared against previously characterized homologs of other organisms, and proteins were examined for the presence of key primary sequence elements. GMDs and GMERs of other organisms are cytosolic soluble enzymes of the short-chain dehydrogenase/reductase (SDR) gene family and feature a Rossman dinucleotide-binding domain (reviewed by [9]; also see references in Table 1). Amino acid alignment of schistosome GMD and GMER to functionally characterized homologs of humans, *Mus*, *Danio*, *Drosophila*, *Caenorhabditis*, *Mortierella*, *Arabidopsis* and *Bacteroides* (listed in Table 1) demonstrated that both genes are well conserved across taxa (30.2% overall identity for GMDs, 13.6% for GMERs) (Figures 3 and 4). In pairwise comparisons, schistosome GMD shares ~53-61% of its primary sequence with homologs (61.2% identical to *Bacteroides* Gmd), while schistosome GMER is ~25-62% identical to its homologs (61.7% identical to human FX). Both GMD and GMER alignments demonstrated the presence of a well-conserved glycine-rich phosphate-binding loop (GxxGxxG; alignment positions 57–63 in GMD; positions 23–29 in GMER), which is key to water-mediated hydrogen bonding between the Rossman fold of redox-associated enzymes and the pyrophosphate of dinucleotide enzyme cofactors (e.g., NAD^+^/NADP^+^) [102], and both enzymes feature the catalytically important SDR-associated [S/T]-Y-K triad (alignment positions 187, 211 and 215 in GMD; positions 126, 155 and 159 in GMER) [103-105]. Additionally, schistosome GMER features conserved cysteine and histidine residues (C-128, H-198) that are thought to be involved in proton exchange between GMER and its epimerization reaction intermediates [105]. Finally, analyses using the Simple Modular Architecture Research Tool (SMART; [106]) and Phobius transmembrane topology and signal peptide prediction server [107] demonstrated that schistosome GMD and GMER lack either a transmembrane domain (TMD) or signal sequence, indicating that both proteins are likely soluble and cytosolic.

**Figure 5:**
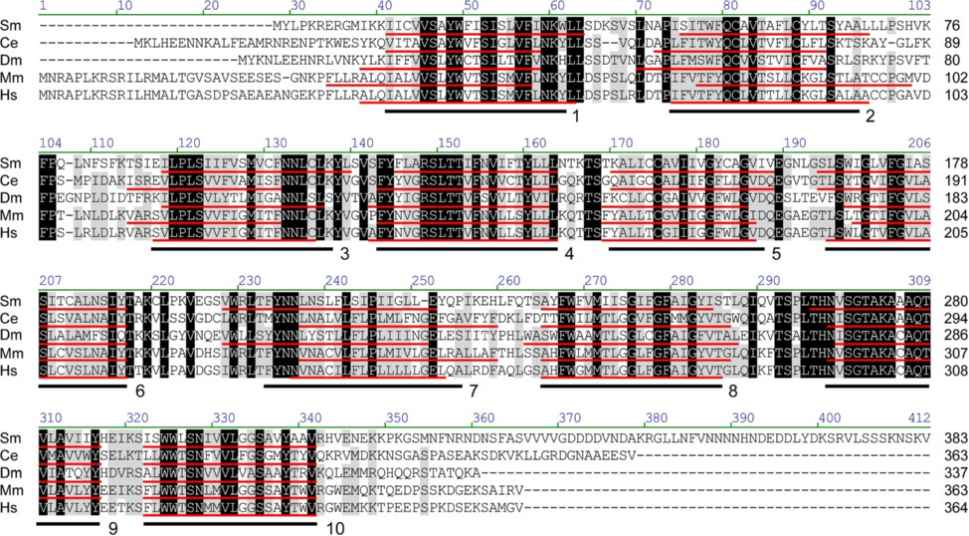
**Amino acid alignment of GDP-L-fucose transporters.** The predicted amino acid sequence of schistosome GFT (Sm) is compared to GFTs of *Caenorhabditis elegans* (Ce), *Drosophila melanogaster* (Dm), *Mus musculus* (Mm) and humans (Hs) (accession numbers in Table 2). Alignment position is indicated above each block, and sequence length is reported to the right of each line. Positions of identity and positions exhibiting at least 80% conservation are highlighted in black and gray, respectively. The positions of ten well-conserved TMDs (underlined below sequences and alignment blocks) were determined using the Phobius transmembrane topology and signal peptide prediction server [107]. Vector NTI Advance 11.0 software alignment settings: BLOSUM45 matrix, gap opening penalty = 12, gap extension penalty = 0.1, gap separation penalty range = 0, no residue-specific or hydrophobic residue gaps.

Previous studies have demonstrated that GFTs are generally Golgi-resident multispan transmembrane proteins with 10 TMDs [27,28,30,70]. Moreover, these genes feature a high degree of conservation across invertebrate and vertebrate taxa. Protein alignments of schistosome GFT with functionally characterized orthologs from *Caenorhabditis*, *Drosophila*, *Mus* and humans (see Table 2) revealed 25.2% overall identity, with pairwise comparisons indicating that schistosome GFT shares ~37-41% identity with orthologous GFTs (Figure 5). A unique feature of the schistosome protein is its conspicuously long C-terminal tail; however, the significance of this extension remains unknown. An analysis of membrane topology using the Phobius server suggested the presence of 10 tightly spaced TMDs, with both N- and C-terminal tails oriented into the cytoplasm (in Additional file 2: Figure S1). For comparison, the positions of the 10 TMDs in known GFTs were also determined using the Phobius server, and alignment with schistosome GFT demonstrated that the spacing of TMDs is roughly conserved across taxa (Figure 5). Altogether, these data support a role for schistosome GFT in GDP-L-fucose transport.

Alternative splice isoforms of GFT that exclude exons 7 and 8 (and the intervening intron) encode a truncated protein featuring 7 TMDs. Importantly, nucleotide-sugar transporters (NSTs), including the GFTs, are part of a diverse drug/metabolite transporter superfamily composed of multispan transmembrane proteins (typically with 4–10 TMDs) that function in drug export, nutrient/metabolite efflux and compartmental metabolite exchange [108,109]. While the observed in-frame deletion of three TMDs likely abolishes GDP-L-fucose transport activity (given it lacks definitive primary sequence characteristics), the truncated GFT could retain its function as an NST (but with altered substrate specificity) or adopt a new class of metabolite transport function altogether. Future studies should assess the biochemical significance of this truncation.

The above topological analyses employed several transmembrane prediction tools (e.g., TMHMM 2.0 and TMpred [110,111]), but Phobius was the only one that predicted all 10 TMDs in most genes. Only TMD 9 of the human GFT was undetected using this method. Lubke *et al*. [27] reported similar difficulty in demonstrating this same TMD, which they attributed to its unusually high hydrophilicity. In general, *in silico* predictions of NST membrane topology are inherently difficult because current algorithms do not account for the relative thinness of the Golgi membrane (~20% thinner) and thus fail to recognize the concomitantly short TMDs of Golgi-resident transmembrane proteins such as NSTs [112]. In fact, at a typical length of 17–22 aa, the TMDs of Golgi proteins are on average five aa shorter than those of plasma membrane-associated proteins [113-115].

### Phylogenetic analysis of nucleotide-sugar transporters

**Figure 6:**
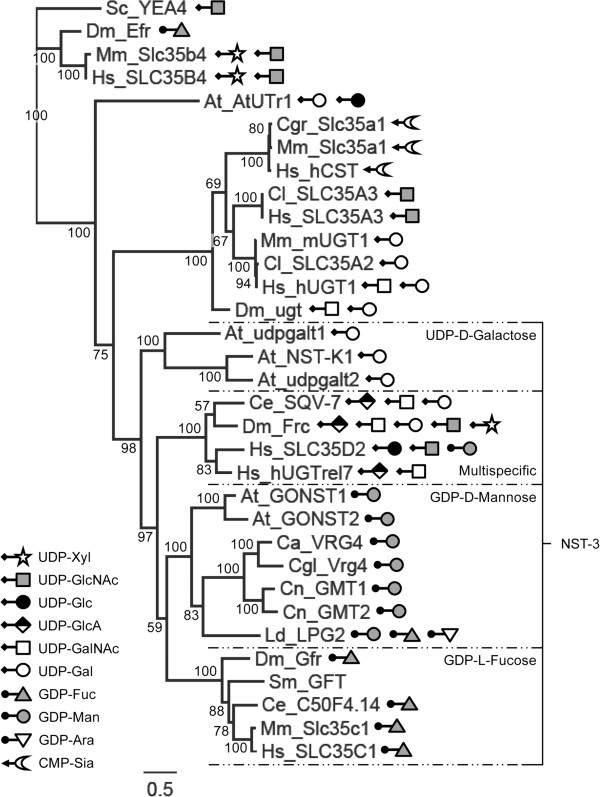
**Phylogenetic tree of nucleotide-sugar transporters.** The amino acid sequences of previously characterized NSTs and putative GFT of *Schistosoma mansoni* (RefSeq/GenBank accession numbers in Table 2) were included in a phylogeny annotated with substrate specificity data. Posterior probabilities are indicated at each node, and genetic divergence (substitutions per site) is represented by the scale bar. Family 3 NSTs [108], which include transporters of GDP-L-fucose, are labeled on the right. To demonstrate the topology of NST families 1 and 2, these data are also presented as a rooted tree in (Additional file 3: Figure S2). UDP-Xyl, UDP-D-xylose; UDP-GlcNAc, UDP-D-N-acetylglucosamine; UDP-Glc, UDP-D-glucose; UDP-GlcA, UDP-D-glucuronic acid; UDP-GalNAc, UDP-D-N-acetylgalactosamine; UDP-Gal, UDP-D-galactose; GDP-Fuc, GDP-L-fucose; GDP-Man, GDP-D-mannose; GDP-Ara, GDP-D-arabinose; CMP-Sia, CMP-sialic acid.

Primary sequence identity alone cannot reliably predict substrate specificity among NST genes [54,112]. NSTs can share as much as 50-60% of their primary sequences and exhibit different substrate specificities while proteins that are only 20% identical can transport the same nucleotide-sugar substrates [63]. However, previous studies have demonstrated that phylogenetic analyses can separate NSTs into functional groups [108,116]. To refine the predicted substrate specificity of schistosome GFT and better understand the structure-function relationship between GFTs and other NSTs, we conducted a phylogenetic analysis of schistosome GFT and a functionally diverse sampling of previously characterized NSTs. The topology of the resultant phylogeny is consistent with observations by Martinez-Duncker *et al*. [108]: the current repertoire of NSTs can be divided into three main families/groups (NST families 1–3), which form separate monophyletic clades (Figure 6; see in Additional file 3: Figure S2 for a rooted tree demonstrating the three NST families). Consistent with the notion that closely related NSTs can be functionally divergent, all three families include members with aberrant substrate specificities. While structure-function relationships in families 1 and 2 remain somewhat unclear, NST family 3 can be broken down into four daughter clades (corresponding to subfamilies J-M in [108]) that correspond to substrate specificity. Subfamily J includes NSTs exhibiting multispecific UDP-sugar transport activities, while subfamilies K, J and M feature NSTs having relatively narrow substrate specificities (GDP-D-mannose, UDP-D-galactose or GDP-L-fucose, respectively). Of the 18 previously characterized family 3 NSTs examined here, only LPG2 of *Leishmania donovani* features uncharacteristic activity for its clade, transporting GDP-L-fucose and GDP-D-arabinose in addition to GDP-D-mannose. Schistosome GFT forms a monophyletic clade with known Golgi-resident GFTs, supporting a predicted role in GDP-L-fucose transport. Notably, *Drosophila* Efr, which delivers GDP-L-fucose to the ER, clusters with NST family 2. This is consistent with other NST family 2 transporters that function in the ER and not the Golgi. Indeed, Martinez-Duncker *et al.*[108] reported that 54% of NST family 2 members feature a C-terminal di-lysine (KKxx) ER-retention/retrieval signal, and one such signal (KKVE) is present in *Drosophila* Efr. In contrast, similar ER-retention/retrieval signals do not exist in schistosome GFT or any of the family 3 NSTs examined here.

### GMD, GMER and GFT mRNA expression in miracidia and primary sporocysts of S. mansoni

**Figure 7:**
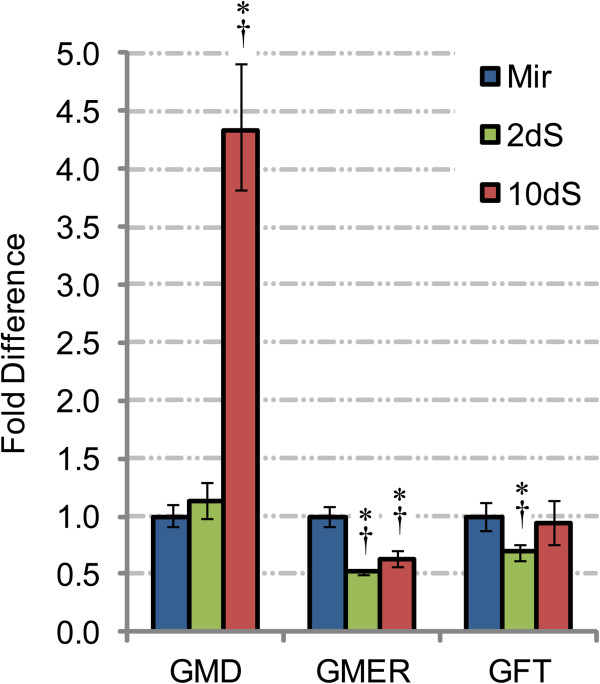
**GDP-L-fucose synthesis- and transport-associated gene transcription in larvae of *Schistosoma mansoni*.** Real-time qPCR was used to examine *GMD*, *GMER* and *GFT* transcription in miracidia (Mir) and 2- and 10-day *in vitro*-cultivated primary sporocysts (2dS and 10dS, respectively). Transcript abundances in primary sporocysts were compared to miracidia (arbitrarily set at 1), and data were analyzed across three biological replicates using heteroscedastic two-sample t- and Wilcoxon rank sum tests, with significance set at *p*≤0.05 (indicated by *) and *p*=0.10 (indicated by †), respectively.

Given recent data demonstrating the abundant expression of fucosylated glycotopes in snail-associated schistosome larvae [7,117] and their predicted immunomodulatory roles in snail hosts, GMD, GMER, and GFT steady-state transcript levels were assayed by qPCR in miracidia and 2- and 10-day *in vitro*-cultivated primary sporocysts. The results indicate that all three genes are differentially expressed during the miracidium-to-primary sporocyst transformation and subsequent cultivation (Figure 7). In conjunction with larval transformation, *GMER* and *GFT* transcript levels declined 48% and 31%, respectively, after two days in culture, while *GMD* expression remained unchanged. During subsequent *in vitro* cultivation of primary sporocysts (up to 10 days), *GMD* transcript abundance climbed ~4-fold while expression of *GMER* and *GFT* stayed the same. These results are somewhat confounding since *GMD* and *GMER* constitute a single biosynthetic pathway.

For comparison, the *S. mansoni* Serial Analysis of Gene Expression (SAGE) Database [96] was examined for relevant SAGE tags, and tags 7188 and 10882 corresponding to *GMD* and *GMER*, respectively, were identified. Consistent with the present study, the SAGE data indicate that *GMD* transcript abundance increases ~3-fold from miracidia to 6-day primary sporocysts while the *GMER*-specific tag 10882 was not detected in either larval stage. Interestingly, both genes exhibited peak expression in 20-day primary sporocysts, suggesting that GDP-L-fucose synthesis potentially increases in older larvae. *GFT* transcript expression (as indicated by tag 4514) followed a similar profile, with relatively low transcript levels in miracidia and 6-day primary sporocysts and peak expression after 20 days in culture. In the present study, if sporocyst cultivation times had been longer, the expression of all three genes may have peaked similarly in older larvae (i.e., >10 days in culture).

The GMDs in bacteria participate in several overlapping synthetic pathways, with reaction intermediates being converted to GDP-L-fucose, GDP-D-rhamnose or GDP-D-talose by GMER, GDP-6-deoxy-d-lyxo-hexos-4-ulose-4-reductase (RMD) and GDP-6-deoxy-D-talose synthetase (GTS), respectively (reviewed in [118]). Additionally, GMDs of *Paramecium bursaria*, *Chlorella* virus 1 and some bacteria (e.g., *Pseudomonas aeruginosa*) are bifunctional, having the added ability to catalyze the same stereospecific reduction as RMD. A similar, still unknown dual functionality or involvement in other biochemical pathways in schistosomes could explain why the observed *GMD* and *GMER* expression profiles vary independently; however, participation of GMD in GDP-D-rhamnose or GDP-D-talose biosynthesis in particular is unlikely because rhamnose and talose, as well as homologs of RMD and GTS, are not observed in *S. mansoni*.

### Recombinant GMD and GMER protein expression, purification, and antibody production

To facilitate analyses of protein expression in larval *S. mansoni*, GMD and GMER were heterologously expressed and purified, and the recombinant proteins were used to raise GMD- and GMER-specific chicken IgY antibodies (in Additional file 4: Figure S3A-B). To assess specificity, antibodies were tested against blotted crude parasite extracts and pure GMD and GMER recombinant antigens. Initially, immunoblots revealed unacceptable levels of crossreactivity (especially between anti-GMD IgY and recombinant GMER; in Additional file 4: Figure S3C), so antibodies were further purified by membrane adsorption against the purified antigens. Subsequent immunoblots demonstrated that antigen specificities of both IgY preparations were greatly improved, showing essentially monospecific reactivities (in Additional file 4: Figure S3D). Membrane-isolated antibodies were used in downstream immunoblot and microscopic analyses.

### Characterization of GMD and GMER protein expression in miracidia and primary sporocysts of S. mansoni

**Figure 8:**
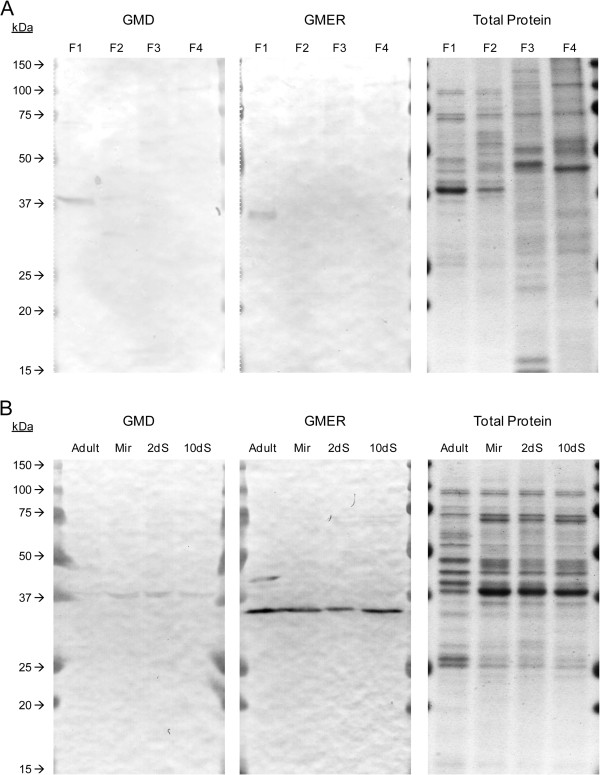
**GMD and GMER protein expression in larval and adult *Schistosoma mansoni*.** Membrane-purified chicken IgY antibodies (see in Additional file 4: Figure S3) were used in western blot analyses of 2-day *in vitro*-cultivated primary sporocyst subcellular protein extracts **(A)**. Four enriched fractions were examined: (F1) cytosol, (F2) membrane/membrane organelle, (F3) nucleus, and (F4) cytoskeleton. Blots indicate the presence of GMD and GMER only in the cytosolic fraction. Next, cytosolic fractions were used to compare GMD and GMER protein expression among mixed-sex adults, miracidia and 2- and 10-day *in vitro*-cultivated primary sporocysts **(B)**. In both experiments, total protein was visualized in-gel by Coomassie staining.

Multiple attempts were made to demonstrate the presence of GMD and GMER in crude adult and larval extracts using western blotting, but only faint bands were produced (Peterson, unpublished data). To enhance detection of GMD and GMER in western blot analyses and concurrently demonstrate their cytosolic localization, 2-day primary sporocysts were serially extracted using a ProteoExtract® Subcellular Proteome Extraction Kit, yielding enriched cytosolic, membrane, nuclear, and cytoskeletal protein fractions. While application of the ProteoExtract® kit for subcellular fractionation of whole schistosome larvae has yet to be experimentally validated regarding the fidelity of differential extraction, Coomassie-stained gels clearly demonstrated compositional differences in the resultant protein fractions and fractionation successfully facilitated detection of GMD and GMER in subsequent immunoblots (Figure 8A). Consistent with their expected roles in cytosolic GDP-L-fucose synthesis, immunoblots revealed the presence of GMD and GMER only in the presumptive cytosolic fraction (bands at 38 and 35 kDa, respectively).

In a comparison of cytosolic extracts from miracidia and 2- and 10-day primary sporocysts, GMD and GMER proteins appear to be stably expressed during larval transformation and subsequent *in vitro* cultivation (Figure 8B). This result seemingly contradicts qPCR and SAGE data described above, which indicate stage-specific differences in *GMD* and *GMER* transcript levels among snail-associated larvae. One possible explanation for the apparent discrepancy between transcript and protein abundances is the inability of qPCR and SAGE approaches to adequately differentiate between “functional” GMD/GMER-coding transcripts and variants that are pretranslationally targeted for nonsense-mediated decay or are translated to truncated proteins not detected by the above methods. For example, while *GMD* gene transcription appears to increase ~4-fold in 10-day *in vitro*-cultivated primary sporocysts, the absolute abundance of “functional” GMD-coding transcripts might remain unchanged, thus resulting in no detectable alteration in protein expression. Additionally, protein turnover rates may be sufficiently low to permit persistence and stable detection regardless of declining transcript abundance (e.g., GMER). Lastly, it should be noted that colorimetric precipitation-mediated detection of immunoreactive proteins is perhaps inadequate for the demonstration of relatively minor differences in protein abundance and application of more quantitative detection methods (e.g., fluorescence) might have revealed low-level stage-specific variations in GMD and GMER expression that mirror the observed changes in gene transcription.

Immunoblots also examined GMD and GMER expression in mixed-sex adult worms (Figure 8B). Cytosolic extracts were seemingly devoid of immunoreactive GMD, suggesting differential expression between adults and larvae. Additionally, adult extracts featured two anti-GMER IgY-reactive bands, one corresponding to GMER and a second at ~42 kDa. The added band potentially represents the translated product of an adult-specific alternative splice isoform; however, none of the observed variants can account for the increased protein size. Alternatively, the band is an artifact of antibody crossreactivity. That adult worms apparently lack GMD while expressing one or more GMER isoforms is confounding, given their roles in the same biosynthetic pathway. One possible explanation is that GMER or an alternative protein isoform has an unknown role in a separate pathway, which drives its expression independent of GMD.

**Figure 9:**
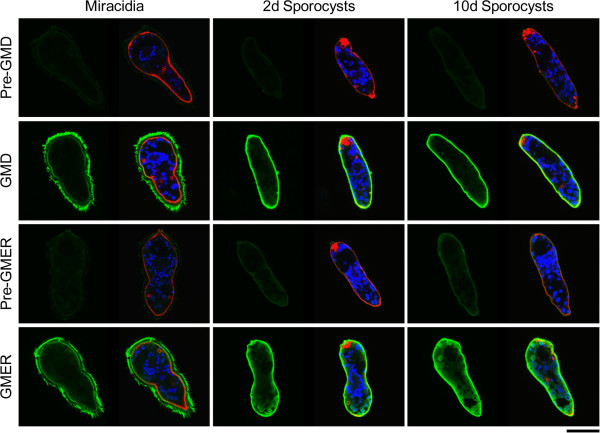
**Localization of *de novo *GDP-L-fucose synthesis in miracidia and primary sporocysts of *Schistosoma mansoni*.** Confocal laser scanning microscopy was used to assess the localization of schistosome GMD and GMER in miracidia and 2- and 10-day *in vitro*-cultivated primary sporocysts. Fixed and permeabilized larvae were immunostained with membrane-purified chicken IgY raised against schistosome GMD and GMER (rows 2 and 4, respectively), and antibody reactivities were assessed relative to control larvae incubated with membrane-adsorbed pre-immune eluates (rows 1 and 3). Panels include paired micrographs depicting GMD/GMER expression (green) alone and merged with counterstained actin (e.g., muscles, flame cells; red) and DNA (e.g., nuclei; blue). Approximate scale is represented in the lower right corner (bar = 50 μm).

Finally, the membrane-purified antibodies were employed in confocal laser scanning microscopy to demonstrate the tissue localization of GMD and GMER proteins in miracidia and 2- and 10-day primary sporocysts (Figure 9). Both proteins were observed predominantly in the ciliated epidermal plates and tegument of miracidia and sporocysts, respectively, while antibodies exhibited at least minor reactivities in internal somatic tissues. Similar patterns of expression in schistosome larvae were observed for several prominent fucosylated glycotopes, including Fucα1-3GalNAcβ1-4GlcNAc (F-LDN) and Fucα1-3GalNAcβ1-4(Fucα1-3)GlcNAc (F-LDN-F) [7]. Importantly, co-localization of schistosome GMD and GMER implies the presence of a complete *de novo* pathway for GDP-L-fucose synthesis, and further supports their roles in fucosylation.

## Conclusions

The present study used a genome-wide homology based bioinformatics approach to identify GDP-L-fucose synthesis- and transport-associated genes in the human blood fluke *Schistosoma mansoni.* The above data indicate that GDP-L-fucose in *S. mansoni* is generated in the cytosol by a *de novo* synthetic pathway comprising GMD and GMER enzymes, after which the resulting activated fucose is imported into the Golgi by the multispan transmembrane protein GFT. Importantly, these enzymes represent a bottleneck in the fucosylation process since GDP-L-fucose is the sole nucleotide-sugar donor utilized by Golgi- and ER-resident FucTs. This research has provided a necessary foundation for future investigations that further explore the role of GDP-L-fucose synthesis and transport in schistosome development and immunobiology. Additionally, the genes identified in this study are potential targets for the development of novel anti-schistosomal chemotherapeutics.

## Abbreviations

Ara: Arabinose; BSA: Bovine serum albumin; CBSS: Chernin’s Balanced Salt Solution; CDS: Coding sequence; FPGT: L-fucose-1-phosphate guanylyltransferase; Fuc: Fucose; FucT: Fucosyltransferase; Fuk: L-Fucokinase; Gal: Galactose; GalNAc: N-acetylgalactosamine; GFT: GDP-L-fucose transporter; Glc: Glucose; GlcA: Glucuronic acid; GlcNAc: N-acetylglucosamine; GMD: GDP-D-mannose-4,6-dehydratase; GMER: GDP-4-keto-6-deoxy-D-mannose-3,5-epimerase-4-reductase; GOI: Gene of interest; LADII: Leukocyte adhesion deficiency type II; Man: Mannose; NCBI: National Center for Biotechnology Information; NST: Nucleotide-sugar transporter; ORF: Open reading frame; PBS: Phosphate-buffered saline; PCB: PreScission™ cleavage buffer; PIC: Protease inhibitor cocktail; PTC: Premature termination codon; qPCR: Quantitative PCR; RACE: Rapid amplification of cDNA ends; RefSeq: Reference Sequence; RT-PCR: Reverse transcription PCR; SAGE: Serial analysis of gene expression; SchistoDB: *Schistosoma mansoni* Database; SDR: Short-chain dehydrogenase/reductase; Sia: Sialic acid; sPBS: Snail PBS; TBS: Tris-buffered saline; TBST: Tris-buffered saline with Tween® 20; TMD: Transmembrane domain; Xyl: Xylose.

## Competing interests

The authors declare that they have no competing interests.

## Authors’ contributions

NAP and TPY designed the above research. NAP and TKA performed experiments and analyzed data. XJW developed the modified subcellular extraction method. NAP wrote the original manuscript, and all authors contributed to its revision and final preparation. All authors have read and approved the manuscript for publication.

## Authors’ information

The above investigation was designed and executed by NAP in partial fulfillment of his doctoral degree in the Comparative Biomedical Sciences program at the University of Wisconsin-Madison under the supervision of TPY. TKA and XJW provided valuable technical expertise in phylogenetic analyses and biochemical methods, respectively.

## Supplementary Material

Additional file 1: Table S1 This PDF document contains Additional file 1: Table S1, which lists the oligonucleotide primers used for RT-PCR, RACE, qPCR and protein expression in this study. Click here for file

Additional file 2: Figure S1 This TIF document contains Additional file 2: Figure S1, which features the results of an *in silico* analysis of GFT membrane topology. Transmembrane domains were identified in the schistosome GFT protein using the Phobius transmembrane topology and signal peptide prediction server [107]. The Phobius output suggested 10 TMDs, a number that is consistent with GDP-L-fucose transporters of other organisms [27,28,30,70] (also see Figure 5) (A). A model based on this output was constructed, portraying the arrangement of the 10 TMDs (numbers indicating the amino acid boundaries of each TMD) as well as the most likely orientation for schistosome GMD within the Golgi membrane (B). Click here for file

Additional file 3: Figure S2 This TIF document contains Additional file 3: Figure S2, which features a rooted phylogenetic tree of nucleotide-sugar transporters (see Figure 6 for detailed unrooted tree). The amino acid sequences of NSTs with previously characterized substrate specificities were obtained from RefSeq and GenBank databases at NCBI (accession numbers in Table 2). A tree was constructed using Bayesian methods implemented in MrBayes v3.12 with mixed amino acid evolutionary models. Monophyletic clades representing NST families 1**–**3 [108] are indicated, and genetic divergence (substitutions per site) is represented by the scale. The tree is rooted on NST family 2. Click here for file

Additional file 4: Figure S3 This TIF document contains Additional file 4: Figure S3, which describes heterologous expression and isolation of recombinant schistosome GMD and GMER proteins and downstream affinity purification of GMD- and GMER-specific polyclonal chicken IgY. GST-GMD and -GMER fusion constructs were created in pGEX-6P-1 vector, and the encoded proteins were expressed in *E. coli*. Fusion protein expression in induced (Ind) and uninduced (Un) cultures was compared by SDS-PAGE fractionation and Coomassie staining of soluble cellular extracts (A). Fusion protein-containing extracts were passed through a GST-affinity column, and bound GMD and GMER were eluted by PreScission™ Protease-mediated cleavage of the GST fusions. Eluates were then analyzed by SDS-PAGE fractionation and Coomassie staining (B). Polyclonal chicken IgY antibodies were raised against recombinant GMD and GMER proteins, and the resultant antibodies were tested by immunoblotting the pure recombinant antigens (C). Due to crossreactivity among the antibodies and antigens (especially between anti-GMD IgY and recombinant GMER), antibodies were affinity-purified by membrane adsorption using bound GMD and GMER antigen. Following elution, antibody preparations were again tested against blots of pure antigen, demonstrating greatly reduced crossreactivity (D). Click here for file

## References

[B1] Hokke CH Deelder AM Hoffmann KF Wuhrer M Glycomics-driven discoveries in schistosome research Exp Parasitol 2007 117 275 283 10.1016/j.exppara.2007.06.003 17659278

[B2] Fitzpatrick JM Peak E Perally S Chalmers IW Barrett J Yoshino TP Ivens AC Hoffmann KF Anti-schistosomal intervention targets identified by lifecycle transcriptomic analyses PLoS Negl Trop Dis 2009 3 e543 10.1371/journal.pntd.0000543 19885392 PMC2764848

[B3] Peterson NA Anderson TK Yoshino TP *In silico* analysis of the fucosylation-associated genome of the human blood fluke *Schistosoma mansoni*: cloning and characterization of the fucosyltransferase multigene family PLoS One 2013 8 e63299 10.1371/journal.pone.0063299 23696810 PMC3655985

[B4] van Remoortere A Hokke CH van Dam GJ van Die I Deelder AM van den Eijnden DH Various stages of *Schistosoma* express Lewis^X^, LacdiNAc, GalNAcβ1-4(Fucα1-3)GlcNAc, and GalNAcβ1-4(Fucα1-2Fucα1-3)GlcNAc carbohydrate epitopes: detection with monoclonal antibodies that are characterized enzymatically synthesized neoglycoproteins Glycobiology 2000 10 601 609 10.1093/glycob/10.6.601 10814702

[B5] Robijn ML Wuhrer M Kornelis D Deelder AM Geyer R Hokke CH Mapping fucosylated epitopes on glycoproteins and glycolipids of *Schistosoma mansoni* cercariae, adult worms, and eggs Parasitology 2005 130 67 77 10.1017/S0031182004006390 15700758

[B6] Wuhrer M Koeleman CA Fitzpatrick JM Hoffmann KF Deelder AM Hokke CH Gender-specific expression of complex-type N-glycans in schistosomes Glycobiology 2006 16 991 1006 10.1093/glycob/cwl020 16825488

[B7] Peterson NA Hokke CH Deelder AM Yoshino TP Glycotope analysis in miracidia and primary sporocysts of *Schistosoma mansoni*: differential expression during the miracidium-to-sporocyst transformation Int J Parasitol 2009 39 1331 1344 10.1016/j.ijpara.2009.06.002 19545571 PMC3740939

[B8] Stanley P Golgi glycosylation Cold Harbor Springs Perspect Biol 2011 3 a005199 10.1101/cshperspect.a005199 PMC3062213 21441588

[B9] Becker DJ Lowe JB Fucose: biosynthesis and biological function in mammals Glycobiology 2003 13 41R 53R 10.1093/glycob/cwg054 12651883

[B10] Yurchenco PD Atkinson PH Equilibration of fucosyl glycoprotein pools in Hela cells Biochemistry 1977 16 944 953 10.1021/bi00624a021 843523

[B11] Rhomberg S Fuchsluger C Rendić D Paschinger K Jantsch V Kosma P Wilson IB Reconstitution *in vitro* of the GDP-fucose biosynthetic pathways of *Caenorhabditis elegans* and *Drosophila melanogaster* FEBS J 2006 273 2244 2256 10.1111/j.1742-4658.2006.05239.x 16650000

[B12] Park SH Pastuszak I Drake R Elbein AD Purification to apparent homogeneity and properties of pig kidney L-fucose kinase J Biol Chem 1998 273 5685 5691 10.1074/jbc.273.10.5685 9488699

[B13] Pastuszak I Ketchum C Hermanson G Sjoberg EJ Drake R Elbein AD GDP-L-fucose pyrophosphorylase. Purification, cDNA cloning, and properties of the enzyme J Biol Chem 1998 273 30165 30174 10.1074/jbc.273.46.30165 9804772

[B14] Hinderlich S Berger M Blume A Chen H Ghaderi D Bauer C Identification of human L-fucose kinase amino acid sequence Biochem Biophys Res Commun 2002 294 650 654 10.1016/S0006-291X(02)00541-7 12056818

[B15] Niittymäki J Mattila P Roos C Huopaniemi L Sjöblom S Renkonen R Cloning and expression of murine enzymes involved in the salvage pathway of GDP-L-fucose Eur J Biochem 2004 271 78 86 14686921 10.1046/j.1432-1033.2003.03904.x

[B16] Coyne MJ Reinap B Lee MM Comstock LE Human symbionts use a host-like pathway for surface fucosylation Science 2005 307 1778 1781 10.1126/science.1106469 15774760

[B17] Kotake T Hojo S Tajima N Matsuoka K Koyama T Tsumuraya Y A bifunctional enzyme with L-fucokinase and GDP-L-fucose pyrophosphorylase activities salvages free L-fucose in *Arabidopsis* J Biol Chem 2008 283 8125 8135 10.1074/jbc.M710078200 18199744

[B18] Roos C Kolmer M Mattila P Renkonen R Composition of *Drosophila melanogaster* proteome involved in fucosylated glycan metabolism J Biol Chem 2002 277 3168 3175 10.1074/jbc.M107927200 11698403

[B19] Caffaro CE Hirschberg CB Nucleotide sugar transporters of the Golgi apparatus: from basic science to diseases Acc Chem Res 2006 39 805 812 10.1021/ar0400239 17115720

[B20] Noda K Miyoshi E Gu J Gao CX Nakahara S Kitada T Honke K Suzuki K Yoshihara H Yoshikawa K Kawano K Tonetti M Kasahara A Hori M Hayashi N Taniguchi N Relationship between elevated FX expression and increased production of GDP-L-fucose, a common donor substrate for fucosylation in human hepatocellular carcinoma and hepatoma cell lines Cancer Res 2003 63 6282 6289 14559815

[B21] Moriwaki K Noda K Nakagawa T Asahi M Yoshihara H Taniguchi N Hayashi N Miyoshi E A high expression of GDP-fucose transporter in hepatocellular carcinoma is a key factor for increases in fucosylation Glycobiology 2007 17 1311 1320 10.1093/glycob/cwm094 17884843

[B22] Niittymäki J Mattila P Renkonen R Differential gene expression of GDP-L-fucose-synthesizing enzymes, GDP-fucose transporter and fucosyltransferase VII APMIS 2006 114 539 548 10.1111/j.1600-0463.2006.apm_461.x 16907860

[B23] Omasa T Tanaka R Doi T Ando M Kitamoto Y Honda K Kishimoto M Ohtake H Decrease in antithrombin III fucosylation by expressing GDP-fucose transporter siRNA in Chinese hamster ovary cells J Biosci Bioeng 2008 106 168 173 10.1263/jbb.106.168 18804060

[B24] Frydman M Etzioni A Eidlitz-Markus T Avidor I Varsano I Shechter Y Orlin JB Gershoni-Baruch R Rambam-Hasharon syndrome of psychomotor retardation, short stature, defective neutrophil motility, and Bombay phenotype Am J Med Genet 1992 44 297 302 10.1002/ajmg.1320440307 1488976

[B25] Etzioni A Frydman M Pollack S Avidor I Phillips ML Paulson JC Gershoni-Baruch R Brief report: recurrent severe infections caused by a novel leukocyte adhesion deficiency N Engl J Med 1992 327 1789 1792 10.1056/NEJM199212173272505 1279426

[B26] Lübke T Marquardt T von Figura K Körner C A new type of carbohydrate-deficient glycoprotein syndrome due to a decreased import of GDP-fucose into the golgi J Biol Chem 1999 274 25986 25989 10.1074/jbc.274.37.25986 10473542

[B27] Lübke T Marquardt T Etzioni A Hartmann E von Figura K Körner C Complementation cloning identifies CDG-IIc, a new type of congenital disorders of glycosylation, as a GDP-fucose transporter deficiency Nat Genet 2001 28 73 76 11326280 10.1038/ng0501-73

[B28] Lühn K Wild MK Eckhardt M Gerardy-Schahn R Vestweber D The gene defective in leukocyte adhesion deficiency II encodes a putative GDP-fucose transporter Nat Genet 2001 28 69 72 11326279 10.1038/ng0501-69

[B29] Helmus Y Denecke J Yakubenia S Robinson P Lühn K Watson DL McGrogan PJ Vestweber D Marquardt T Wild MK Leukocyte adhesion deficiency II patients with a dual defect of the GDP-fucose transporter Blood 2006 107 3959 3966 10.1182/blood-2005-08-3334 16455955

[B30] Hellbusch CC Sperandio M Frommhold D Yakubenia S Wild MK Popovici D Vestweber D Gröne HJ von Figura K Lübke T Körner C Golgi GDP-fucose transporter-deficient mice mimic congenital disorder of glycosylation IIc/leukocyte adhesion deficiency II J Biol Chem 2007 282 10762 10772 10.1074/jbc.M700314200 17276979

[B31] Yoshino TP Laursen JR Production of *Schistosoma mansoni* daughter sporocysts from mother sporocysts maintained in synxenic culture with *Biomphalaria glabrata* embryonic (Bge) cells J Parasitol 1995 81 714 722 10.2307/3283960 7472861

[B32] Nolan LE Carriker JP Observations on the biology of the snail *Lymnaea stagnalis appressa* during twenty years of laboratory culture Am Midl Nat 1946 36 467 493 10.2307/2421516

[B33] Chernin E Observations on hearts explanted *in vitro* from the snail *Australorbis glabratus* J Parasitol 1963 49 353 364 10.2307/3275797 14020610

[B34] Altschul SF Madden TL Schaffer AA Zhang J Zhang Z Miller W Lipman DJ Gapped BLAST and PSI-BLAST: a new generation of protein database search programs Nucleic Acids Res 1997 25 3389 3402 10.1093/nar/25.17.3389 9254694 PMC146917

[B35] Zerlotini A Heiges M Wang H Moraes RL Dominitini AJ Ruiz JC Kissinger JC Oliveira G SchistoDB: a *Schistosoma mansoni* genome resource Nucleic Acids Res 2009 37 D579 D582 10.1093/nar/gkn681 18842636 PMC2686589

[B36] Ohyama C Smith PL Angata K Fukuda MN Lowe JB Fukuda M Molecular cloning and expression of GDP-D-mannose-4,6-dehydratase, a key enzyme for fucose metabolism defective in Lec13 cells J Biol Chem 1998 273 14582 14587 10.1074/jbc.273.23.14582 9603974

[B37] Sullivan FX Kumar R Kriz R Stahl M Xu GY Rouse J Chang XJ Boodhoo A Potvin B Cumming DA Molecular cloning of human GDP-mannose 4,6-dehydratase and reconstitution of GDP-fucose biosynthesis *in vitro* J Biol Chem 1998 273 8193 8202 10.1074/jbc.273.14.8193 9525924

[B38] Bisso A Sturla L Zanardi D De Flora A Tonetti M Structural and enzymatic characterization of human recombinant GDP-D-mannose-4,6-dehydratase FEBS Lett 1999 456 370 374 10.1016/S0014-5793(99)00982-5 10462046

[B39] Imai-Nishiya H Mori K Inoue M Wakitani M Iida S Shitara K Satoh M Double knockdown of α1,6-fucosyltransferase (FUT8) and GDP-mannose 4,6-dehydratase (GMD) in antibody-producing cells: a new strategy for generating fully non-fucosylated therapeutic antibodies with enhanced ADCC BMC Biotechnol 2007 7 84 10.1186/1472-6750-7-84 18047682 PMC2216013

[B40] Tonetti M Sturla L Bisso A Benatti U De Flora A Synthesis of GDP-L-fucose by the human FX protein J Biol Chem 1996 271 27274 27279 10.1074/jbc.271.44.27274 8910301

[B41] Zipin A Israeli-Amit M Meshel T Sagi-Assif O Yron I Lifshitz V Bacharach E Smorodinsky NI Many A Czernilofsky PA Morton DL Witz IP Tumor-microenvironment interactions: the fucose-generating FX enzyme controls adhesive properties of colorectal cancer cells Cancer Res 2004 64 6571 6578 10.1158/0008-5472.CAN-03-4038 15374970

[B42] Quirk S Seley KL Substrate discrimination by the human GTP fucose pyrophosphorylase Biochemistry 2005 44 10854 10863 10.1021/bi0503605 16086588

[B43] Quirk S Seley KL Identification of catalytic amino acids in the human GTP fucose pyrophosphorylase active site Biochemistry 2005 44 13172 13178 10.1021/bi051288d 16185085

[B44] Smith PL Myers JT Rogers CE Zhou L Petryniak B Becker DJ Homeister JW Lowe JB Conditional control of selectin ligand expression and global fucosylation events in mice with a targeted mutation at the FX locus J Cell Biol 2002 158 801 815 10.1083/jcb.200203125 12186857 PMC2174027

[B45] Becker DJ Myers JT Ruff MM Smith PL Gillespie BW Ginsburg DW Lowe JB Strain-specific modification of lethality in fucose-deficient mice Mamm Genome 2003 14 130 139 10.1007/s00335-002-2212-5 12584608

[B46] Ohata S Kinoshita S Aoki R Tanaka H Wada H Tsuruoka-Kinoshita S Tsuboi T Watabe S Okamoto H Neuroepithelial cells require fucosylated glycans to guide the migration of vagus motor neuron progenitors in the developing zebrafish hindbrain Development 2009 136 1653 1663 10.1242/dev.033290 19369395

[B47] Song Y Willer JR Scherer PC Panzer JA Kugath A Skordalakes E Gregg RG Willer GB Balice-Gordon RJ Neural and synaptic defects in slytherin, a zebrafish model for human congenital disorders of glycosylation PLoS One 2010 5 e13743 10.1371/journal.pone.0013743 21060795 PMC2966427

[B48] Ren Y Perepelov AV Wang H Zhang H Knirel YA Wang L Chen W Biochemical characterization of GDP-L-fucose *de novo* synthesis pathway in fungus *Mortierella alpina* Biochem Biophys Res Commun 2010 391 1663 1669 10.1016/j.bbrc.2009.12.116 20035716

[B49] Bonin CP Freshour G Hahn MG Vanzin GF Reiter WD The GMD1 and GMD2 genes of *Arabidopsis* encode isoforms of GDP-D-mannose 4,6-dehydratase with cell type-specific expression patterns Plant Physiol 2003 132 883 892 10.1104/pp.103.022368 12805618 PMC167028

[B50] Bonin CP Potter I Vanzin GF Reiter WD The MUR1 gene of *Arabidopsis thaliana* encodes an isoform of GDP-D-mannose-4,6-dehydratase, catalyzing the first step in the *de novo* synthesis of GDP-L-fucose Proc Natl Acad Sci USA 1997 94 2085 2090 10.1073/pnas.94.5.2085 9050909 PMC20047

[B51] Bonin CP Reiter WD A bifunctional epimerase-reductase acts downstream of the MUR1 gene product and completes the *de novo* synthesis of GDP-L-fucose in *Arabidopsis* Plant J 2000 21 445 454 10.1046/j.1365-313x.2000.00698.x 10758496

[B52] Nakayama K Maeda Y Jigami Y Interaction of GDP-4-keto-6-deoxymannose-3,5-epimerase-4-reductase with GDP-mannose-4,6-dehydratase stabilizes the enzyme activity for formation of GDP-fucose from GDP-mannose Glycobiology 2003 13 673 680 10.1093/glycob/cwg099 12881408

[B53] Wang W Hu T Frantom PA Zheng T Gerwe B Del Amo DS Garret S Seidel RD 3rd Wu P Chemoenzymatic synthesis of GDP-L-fucose and the Lewis X glycan derivatives Proc Natl Acad Sci USA 2009 106 16096 16101 10.1073/pnas.0908248106 19805264 PMC2752511

[B54] Hirschberg CB Golgi nucleotide sugar transport and leukocyte adhesion deficiency II J Clin Invest 2001 108 3 6 11435449 10.1172/JCI13480 PMC209350

[B55] Ashikov A Routier F Fuhlrott J Helmus Y Wild M Gerardy-Schahn R Bakker H The human solute carrier gene SLC35B4 encodes a bifunctional nucleotide sugar transporter with specificity for UDP-xylose and UDP-N-acetylglucosamine J Biol Chem 2005 280 27230 27235 10.1074/jbc.M504783200 15911612

[B56] Ishida N Yoshioka S Chiba Y Takeuchi M Kawakita M Molecular cloning and functional expression of the human Golgi UDP-N-acetylglucosamine transporter J Biochem 1999 126 68 77 10.1093/oxfordjournals.jbchem.a022437 10393322

[B57] Suda T Kamiyama S Suzuki M Kikuchi N Nakayama K Narimatsu H Jigami Y Aoki T Nishihara S Molecular cloning and characterization of a human multisubstrate specific nucleotide-sugar transporter homologous to *Drosophila* fringe connection J Biol Chem 2004 279 26469 26474 10.1074/jbc.M311353200 15082721

[B58] Muraoka M Kawakita M Ishida N Molecular characterization of human UDP-glucuronic acid/UDP-N-acetylgalactosamine transporter, a novel nucleotide sugar transporter with dual substrate specificity FEBS Lett 2001 495 87 93 10.1016/S0014-5793(01)02358-4 11322953

[B59] Miura N Ishida N Hoshino M Yamauchi M Hara T Ayusawa D Kawakita M Human UDP-galactose translocator: molecular cloning of a complementary DNA that complements the genetic defect of a mutant cell line deficient in UDP-galactose translocator J Biochem 1996 120 236 241 10.1093/oxfordjournals.jbchem.a021404 8889805

[B60] Aoki K Ishida N Kawakita M Substrate recognition by UDP-galactose and CMP-sialic acid transporters. Different sets of transmembrane helices are utilized for the specific recognition of UDP-galactose and CMP-sialic acid J Biol Chem 2001 276 21555 21561 10.1074/jbc.M101462200 11279205

[B61] Aoki K Ishida N Kawakita M Substrate recognition by nucleotide sugar transporters: further characterization of substrate recognition regions by analyses of UDP-galactose/CMP-sialic acid transporter chimeras and biochemical analysis of the substrate specificity of parental and chimeric transporters J Biol Chem 2003 278 22887 22893 10.1074/jbc.M302620200 12682060

[B62] Segawa H Kawakita M Ishida N Human and *Drosophila* UDP-galactose transporters transportUDP-N-acetylgalactosamine in addition to UDP-galactose Eur J Biochem 2002 269 128 138 10.1046/j.0014-2956.2001.02632.x 11784306

[B63] Guillen E Abeijon C Hirschberg CB Mammalian Golgi apparatus UDP-N-acetylglucosamine transporter: molecular cloning by phenotypic correction of a yeast mutant Proc Natl Acad Sci USA 1998 95 7888 7892 10.1073/pnas.95.14.7888 9653110 PMC20899

[B64] Olczak M Guillen E Characterization of a mutation and an alternative splicing of UDP-galactose transporter in MDCK-RCAr cell line Biochim Biophys Acta 2006 1763 82 92 10.1016/j.bbamcr.2005.12.006 16434112

[B65] Yakubenia S Frommhold D Schölch D Hellbusch CC Körner C Petri B Jones C Ipe U Bixel MG Krempien R Sperandio M Wild MK Leukocyte trafficking in a mouse model for leukocyte adhesion deficiency II/congenital disorder of glycosylation IIc Blood 2008 112 1472 1481 10.1182/blood-2008-01-132035 18541720

[B66] Yazbek SN Buchner DA Geisinger JM Burrage LC Spiezio SH Zentner GE Hsieh CW Scacheri PC Croniger CM Nadeau JH Deep congenic analysis identifies many strong, context-dependent QTLs, one of which, Slc35b4, regulates obesity and glucose homeostasis Genome Res 2011 21 1065 1073 10.1101/gr.120741.111 21507882 PMC3129249

[B67] Ishida N Yoshioka S Iida M Sudo K Miura N Aoki K Kawakita M Indispensability of transmembrane domains of Golgi UDP-galactose transporter as revealed by analysis of genetic defects in UDP-galactose transporter-deficient murine had-1 mutant cell lines and construction of deletion mutants J Biochem 1999 126 1107 1117 10.1093/oxfordjournals.jbchem.a022556 10578063

[B68] Maggioni A von Itzstein M Gerardy-Schahn R Tiralongo J Targeting the expression of functional murine CMP-sialic acid transporter to the *E. coli* inner membrane Biochem Biophys Res Commun 2007 362 779 784 10.1016/j.bbrc.2007.08.070 17764658

[B69] Eckhardt M Gerardy-Schahn R Molecular cloning of the hamster CMP-sialic acid transporter Eur J Biochem 1997 248 187 192 10.1111/j.1432-1033.1997.00187.x 9310377

[B70] Lühn K Laskowska A Pielage J Klämbt C Ipe U Vestweber D Wild MK Identification and molecular cloning of a functional GDP-fucose transporter in *Drosophila melanogaster* Exp Cell Res 2004 301 242 250 10.1016/j.yexcr.2004.08.043 15530860

[B71] Ishikawa HO Higashi S Ayukawa T Sasamura T Kitagawa M Harigaya K Aoki K Ishida N Sanai Y Matsuno K Notch deficiency implicated in the pathogenesis of congenital disorder of glycosylation IIc Proc Natl Acad Sci USA 2005 102 18532 18537 10.1073/pnas.0504115102 16344471 PMC1317902

[B72] Ishikawa HO Ayukawa T Nakayama M Higashi S Kamiyama S Nishihara S Aoki K Ishida N Sanai Y Matsuno K Two pathways for importing GDP-fucose into the endoplasmic reticulum lumen function redundantly in the O-fucosylation of Notch in *Drosophila* J Biol Chem 2010 285 122 4129 10.1074/jbc.M109.016964 PMC2823552 19948734

[B73] Goto S Taniguchi M Muraoka M Toyoda H Sado Y Kawakita M Hayashi S UDP-sugar transporter implicated in glycosylation and processing of Notch Nat Cell Biol 2001 3 816 822 10.1038/ncb0901-816 11533661

[B74] Selva EM Hong K Baeg GH Beverley SM Turco SJ Perrimon N Häcker U Dual role of the fringe connection gene in both heparan sulphate and fringe-dependent signalling events Nat Cell Biol 2001 3 809 815 10.1038/ncb0901-809 11533660

[B75] Aumiller JJ Jarvis DL Expression and functional characterization of a nucleotide sugar transporter from *Drosophila melanogaster*: relevance to protein glycosylation in insect cell expression systems Protein Expr Purif 2002 26 438 448 10.1016/S1046-5928(02)00550-8 12460768 PMC3641582

[B76] Berninsone P Hwang HY Zemtseva I Horvitz HR Hirschberg CB SQV-7, a protein involved in *Caenorhabditis elegans* epithelial invagination and early embryogenesis, transports UDP-glucuronic acid, UDP-N-acetylgalactosamine, and UDP-galactose Proc Natl Acad Sci USA 2001 98 3738 3743 10.1073/pnas.061593098 11259660 PMC31122

[B77] Hong K Ma D Beverley SM Turco SJ The *Leishmania* GDP-mannose transporter is an autonomous, multi-specific, hexameric complex of LPG2 subunits Biochemistry 2000 39 2013 2022 10.1021/bi992363l 10684651

[B78] Cottrell TR Griffith CL Liu H Nenninger AA Doering TL The pathogenic fungus *Cryptococcus neoformans* expresses two functional GDP-mannose transporters with distinct expression patterns and roles in capsule synthesis Eukaryot Cell 2007 6 776 785 10.1128/EC.00015-07 17351078 PMC1899245

[B79] Roy SK Chiba Y Takeuchi M Jigami Y Characterization of Yeast Yea4p, a uridine diphosphate-N-acetylglucosamine transporter localized in the endoplasmic reticulum and required for chitin synthesis J Biol Chem 2000 275 13580 13587 10.1074/jbc.275.18.13580 10788474

[B80] Nishikawa A Poster JB Jigami Y Dean N Molecular and phenotypic analysis of CaVRG4, encoding an essential Golgi apparatus GDP-mannose transporter J Bacteriol 2002 184 29 42 10.1128/JB.184.1.29-42.2002 11741841 PMC134776

[B81] Nishikawa A Mendez B Jigami Y Dean N Identification of a *Candida glabrata* homologue of the *S. cerevisiae* VRG4 gene, encoding the Golgi GDP-mannose transporter Yeast 2002 19 691 698 10.1002/yea.854 12185838

[B82] Baldwin TC Handford MG Yuseff MI Orellana A Dupree P Identification and characterization of GONST1, a golgi-localized GDP-mannose transporter in *Arabidopsis* Plant Cell 2001 13 2283 2295 11595802 10.1105/tpc.010247 PMC139159

[B83] Handford MG Sicilia F Brandizzi F Chung JH Dupree P *Arabidopsis thaliana* expresses multiple Golgi-localised nucleotide-sugar transporters related to GONST1 Mol Genet Genomics 2004 272 397 410 10.1007/s00438-004-1071-z 15480787

[B84] Norambuena L Marchant L Berninsone P Hirschberg CB Silva H Orellana A Transport of UDP-galactose in plants. Identification and functional characterization of AtUTr1, an *Arabidopsis thaliana* UDP-galactos/UDP-glucose transporter J Biol Chem 2002 277 32923 32929 10.1074/jbc.M204081200 12042319

[B85] Rollwitz I Santaella M Hille D Flügge UI Fischer K Characterization of AtNST-KT1, a novel UDP-galactosetransporter from *Arabidopsis thaliana* FEBS Lett 2006 580 4246 4251 10.1016/j.febslet.2006.06.082 16831428

[B86] Bakker H Routier F Oelmann S Jordi W Lommen A Gerardy-Schahn R Bosch D Molecular cloning of two *Arabidopsis* UDP-galactose transporters by complementation of a deficient Chinese hamster ovary cell line Glycobiology 2005 15 193 201 15456736 10.1093/glycob/cwh159

[B87] Owczarzy R Tataurov AV Wu Y Manthey JA McQuisten KA Almabrazi HG Pedersen KF Lin Y Garretson J McEntaggart NO Sailor CA Dawson RB Peek AS IDT SciTools: a suite for analysis and design of nucleic acid oligomers Nucleic Acids Res 2008 36 W163 W169 10.1093/nar/gkn198 18440976 PMC2447751

[B88] Edgar RC MUSCLE: multiple sequence alignment with high accuracy and high throughput Nucleic Acids Res 2004 32 1792 1797 10.1093/nar/gkh340 15034147 PMC390337

[B89] Maddison WP Maddison DR Mesquite: a modular system for evolutionary analysis. Version 2.72 http://mesquiteproject.org

[B90] Price MN Dehal PS Arkin AP FastTree 2–approximately maximum-likelihood trees for large alignments PLoS One 2010 5 e9490 10.1371/journal.pone.0009490 20224823 PMC2835736

[B91] Ronquist F Huelsenbeck JP MrBayes 3: Bayesian phylogenetic inference under mixed models Bioinformatics 2003 19 1572 1574 10.1093/bioinformatics/btg180 12912839

[B92] Huelsenbeck JP Bollback JP Empirical and hierarchical Bayesian estimation of ancestral states Syst Biol 2001 50 351 366 12116580

[B93] Drummond AJ Rambaut A BEAST: Bayesian evolutionary analysis by sampling trees BMC Evol Biol 2007 7 214 10.1186/1471-2148-7-214 17996036 PMC2247476

[B94] Nylander JAA Wilgenbusch JC Warren DL Swofford DL AWTY (are we there yet?): a system for graphical exploration of MCMC convergence in Bayesian phylogenetics Bioinformatics 2008 24 581 583 10.1093/bioinformatics/btm388 17766271

[B95] Applied Biosystems Guide to performing relative quantitation of gene expression using real-time quantitative PCR 2008 Foster City, CA

[B96] Williams DL Sayed AA Bernier J Birkeland SR Cipriano MJ Papa AR McArthur AG Taft A Vermeire JJ Yoshino TP Profiling *Schistosoma mansoni* development using serial analysis of gene expression (SAGE) Exp Parasitol 2007 117 246 258 10.1016/j.exppara.2007.05.001 17577588 PMC2121609

[B97] Bustin SA Benes V Garson JA Hellemans J Huggett J Kubista M Mueller R Nolan T Pfaffl MW Shipley GL Vandesompele J Wittwer CT The MIQE guidelines: minimum information for publication of quantitative real-time PCR experiments Clin Chem 2009 55 611 622 10.1373/clinchem.2008.112797 19246619

[B98] Kalsotra A Cooper TA Functional consequences of developmentally regulated alternative splicing Nat Rev Genet 2011 12 715 729 21921927 10.1038/nrg3052 PMC3321218

[B99] Lantner F Ziv E Ram D Schechter I Different forms of the mRNA encoding the heat-shock transcription factor are expressed during the life cycle of the parasitic helminth *Schistosoma mansoni* Eur J Biochem 1998 253 390 398 10.1046/j.1432-1327.1998.2530390.x 9654088

[B100] Ram D Ziv E Lantner F Lardans V Schechter I Stage-specific alternative splicing of the heat-shock transcription factor during the life-cycle of *Schistosoma mansoni* Parasitology 2004 129 587 596 10.1017/S003118200400602X 15552403

[B101] DeMarco R Oliveira KC Venancio TM Verjovski-Almeida S Gender biased differential alternative splicing patterns of the transcriptional cofactor CA150 gene in *Schistosoma mansoni* Mol Biochem Parasitol 2007 150 123 131 16904200 10.1016/j.molbiopara.2006.07.002

[B102] Bottoms CA Smith PE Tanner JJ A structurally conserved water molecule in Rossman dinucleotide-binding domains Protein Sci 2002 11 2125 2137 12192068 10.1110/ps.0213502 PMC2373605

[B103] Somoza JR Menon S Schmidt H Joseph-McCarthy D Dessen A Stahl ML Somers WS Sullivan FX Structural and kinetic analysis of *Escherichia coli* GDP-mannose 4,6 dehydratase provides insights into the enzyme's catalytic mechanism and regulation by GDP-fucose Structure 2000 8 123 135 10.1016/S0969-2126(00)00088-5 10673432

[B104] Mulichak AM Bonin CP Reiter WD Garavito RM Structure of the MUR1 GDP-mannose 4,6-dehydratase from *Arabidopsis thaliana*: implications for ligand binding and specificity Biochemistry 2002 41 15578 15589 10.1021/bi0266683 12501186

[B105] Rosano C Bisso A Izzo G Tonetti M Sturla L De Flora A Bolognesi M Probing the catalytic mechanism of GDP-4-keto-6-deoxy-d-mannose Epimerase/Reductase by kinetic and crystallographic characterization of site-specific mutants J Mol Biol 2000 303 77 91 10.1006/jmbi.2000.4106 11021971

[B106] Schultz J Milpetz F Bork P Ponting CP SMART, a simple modular architecture research tool: identification of signaling domains Proc Natl Acad Sci USA 1998 95 5857 5864 10.1073/pnas.95.11.5857 9600884 PMC34487

[B107] Käll L Krogh A Sonnhammer EL Advantages of combined transmembrane topology and signal peptide prediction–the Phobius web server Nucleic Acids Res 2007 35 W429 W432 10.1093/nar/gkm256 17483518 PMC1933244

[B108] Martinez-Duncker I Mollicone R Codogno P Oriol R The nucleotide-sugar transporter family: a phylogenetic approach Biochimie 2003 85 245 260 10.1016/S0300-9084(03)00046-4 12770764

[B109] Jack DL Yang NM Saier MH Jr The drug/metabolite transporter superfamily Eur J Biochem 2001 268 3620 3639 10.1046/j.1432-1327.2001.02265.x 11432728

[B110] Sonnhammer ELL von Heijne G Krogh A Glasgow J, Littlejohn T, Major F, Lathrop R, Sankoff D, Sensen C A hidden Markov model for predicting transmembrane helices in protein sequences Procedings of the Sixth International Conference on Intelligent Systems for Molecular Biology: 28 June-1 July, 1998; Montréal, Québec, Canada 1998 Menlo Park, CA: Association for the Advancement of Artificial Intelligence Press 175 182 9783223

[B111] Hofmann K Stoffel W TMbase - A database of membrane spanning proteins segments Biol Chem Hoppe Seyler 1993 347 166

[B112] Berninsone PM Hirschberg CB Nucleotide sugar transporters of the Golgi apparatus Curr Opin Struct Biol 2000 10 542 547 10.1016/S0959-440X(00)00128-7 11042451

[B113] Munro S A comparison of the transmembrane domains of Golgi and plasma membrane proteins Biochem Soc Trans 1995 23 527 530 8566407 10.1042/bst0230527

[B114] Yuan Z Teasdale RD Prediction of Golgi Type II membrane proteins based on their transmembrane domains Bioinformatics 2002 18 1109 1115 10.1093/bioinformatics/18.8.1109 12176834

[B115] Sharpe HJ Stevens TJ Munro S A comprehensive comparison of transmembrane domains reveals organelle-specific properties Cell 2010 142 158 169 10.1016/j.cell.2010.05.037 20603021 PMC2928124

[B116] Handford M Rodriguez-Furlán C Orellana A Nucleotide-sugar transporters: structure, function and roles *in vivo* Braz J Med Biol Res 2006 39 1149 1158 10.1590/S0100-879X2006000900002 16981043

[B117] Lehr T Beuerlein K Doenhoff MJ Grevelding CG Geyer R Localization of carbohydrate determinants common to *Biomphalaria glabrata* as well as to sporocysts and miracidia of *Schistosoma mansoni* Parasitology 2008 135 931 942 18507884 10.1017/S0031182008004514

[B118] Mäki M Renkonen R Biosynthesis of 6-deoxyhexose glycans in bacteria Glycobiology 2004 14 1R 15R 10.1093/glycob/cwh054 14693916

