# Supplementary material for: In silico analysis of the fucosylation-associated genome of the human blood fluke Schistosoma mansoni: cloning and characterization of the enzymes involved in GDP-L-fucose synthesis and Golgi import
Source: Parasit Vectors. 2013 Jul 9;6:201. doi: 10.1186/1756-3305-6-201 (PMC3718619; doi:10.1186/1756-3305-6-201)
Supplement: Additional file 1: Table S1 — This PDF document contains Additional file 1: Table S1, which lists the oligonucleotide primers used for RT-PCR, RACE, qPCR and protein expression in this study. [file 1756-3305-6-201-S1.pdf]

Supplementary Table S1. List of primers used in this study for RT-PCR, RACE, qPCR, and protein expression

A. Primers for RT-PCR confirmation of gene transcription

Gene	Forward primer	Reverse primer
GMD	5′-TTAGCTGCTCAGTCCCATGTGAAG-3′	5′-CTCCGGTGTATCACGTTGAAGCAT-3′
GMER	5′-AGCGCATACGCCCAACCTATGTAA-3′	5′-ACTGACGCAATGGCTTACCAGAAC-3′
GFT	5′-CGTATGCAGCTCTGCTGTTACCTAG-3′	5′-CATGTCGAACAGCTGCATAAACTGC-3′

B. Primers for 5′ and 3′ RACE of gene transcripts

Gene	Forward primer (3′ RACE)	Reverse primer (5′ RACE)
GMD	5′-ACCCGAGGTCACCGTACAGCGTTGCG-3′	5′-ACGTTTCTCCTCGCCGAGGGCTTTCATGA-3′
	5′-TCATGAAAGCCCTCGGCGAGGAGAAACGT-3′	5′-CGCAACGCTGTACGGTGACCTCGGGT-3′
GMER	5′-CCACTTAGCTGCCAAAGTCGGCGGTTTG-3′	5′-ACTGACGCAATGGCTTACCAGAACCCCA-3′
	5′-TGGGGTTCTGGTAAGCCATTGCGTCAGT-3′	5′-CAAACCGCCGACTTTGGCAGCTAAGTGG-3′
GFT	5′-TCCGGTACTGCTAAGGCAGCTGCACA-3′	5′-TGTGCAGCTGCCTTAGCAGTACCGGA-3′
	5′-GCGGATCAGCAGTTTATGCAGCTGTTCGAC-3′	5′-TCACATGACTAGGTAACAGCAGAGCTGCA-3′

C. Primers for RT-PCR amplification of CDS regions of gene transcripts

Gene	Forward primer	Reverse primer
GMD	5′-CTTGGCAATGAACAATAACGGTG-3′	5′-TCAGACTAAATTGGGAAAGCAGATTG-3′
GMER	5′-TTCTAGGGCCTATATATCCAGCAG-3′	5′-CTAGACACAACTAGCACCGAATAG-3′
GFT	5′-GTATAGAGCGTTGCCAATGAGG-3′	5′-CAAGTTATTAAGAAACCCATAGAGAGC-3′

D. Primers for real-time qPCR analyses of gene transcript expression, including the calibrator GAPDH used in RNAi experiments

Gene	Forward primer	Reverse primer
GMD	5′-GCAATGAACAATAACGGTGTGAG-3′	5′-AGAACTCGGCCAAATAAGAGC-3′
GMER	5′-GAAATTTCGATCCGTCAAGCTG-3′	5′-ACCATCCGCTTTAGTTGTGTC-3′
GFT	5′-AGTCTGTATCGCTAAATGCACC-3′	5′-ACATGACTAGGTAACAGCAGAG-3′
ATPsf	5′-GAATACAATGCACGAGTACATGG-3′	5′-CTACGTGAAAACCACTCACTG-3′
GroES	5′-AAGTCGTACTGGACGAAAATGAG-3′	5′-GGACTAGATCTAAAACACCGGTC-3′

E. BamHI (ggatcc)/EcoRI (gaattc)-tagged primers for preparation of GMD and GMER expression constructs (gene-specific sequences underlined)

Gene	Forward primer (BamHI-tagged)	Reverse primer (BamHI/EcoRI-tagged)
GMD	5′-cctgggatccATGAACAATAACGGTGTGAGTTGTG-3′	5′-acctggaattcGCAGATTGAAAGTTAGTTAAACAGGAG-3′
GMER	5′-cctgggatccATGGAAACCACTGTATTAGTTACAGG-3′	5′-cctgggatccCTAGACACAACTAGCACCGAATAG-3′
