# Supplementary figures and images for: In silico analysis of the fucosylation-associated genome of the human blood fluke Schistosoma mansoni: cloning and characterization of the enzymes involved in GDP-L-fucose synthesis and Golgi import
Source: Parasit Vectors. 2013 Jul 9;6:201. doi: 10.1186/1756-3305-6-201 (PMC3718619; doi:10.1186/1756-3305-6-201)

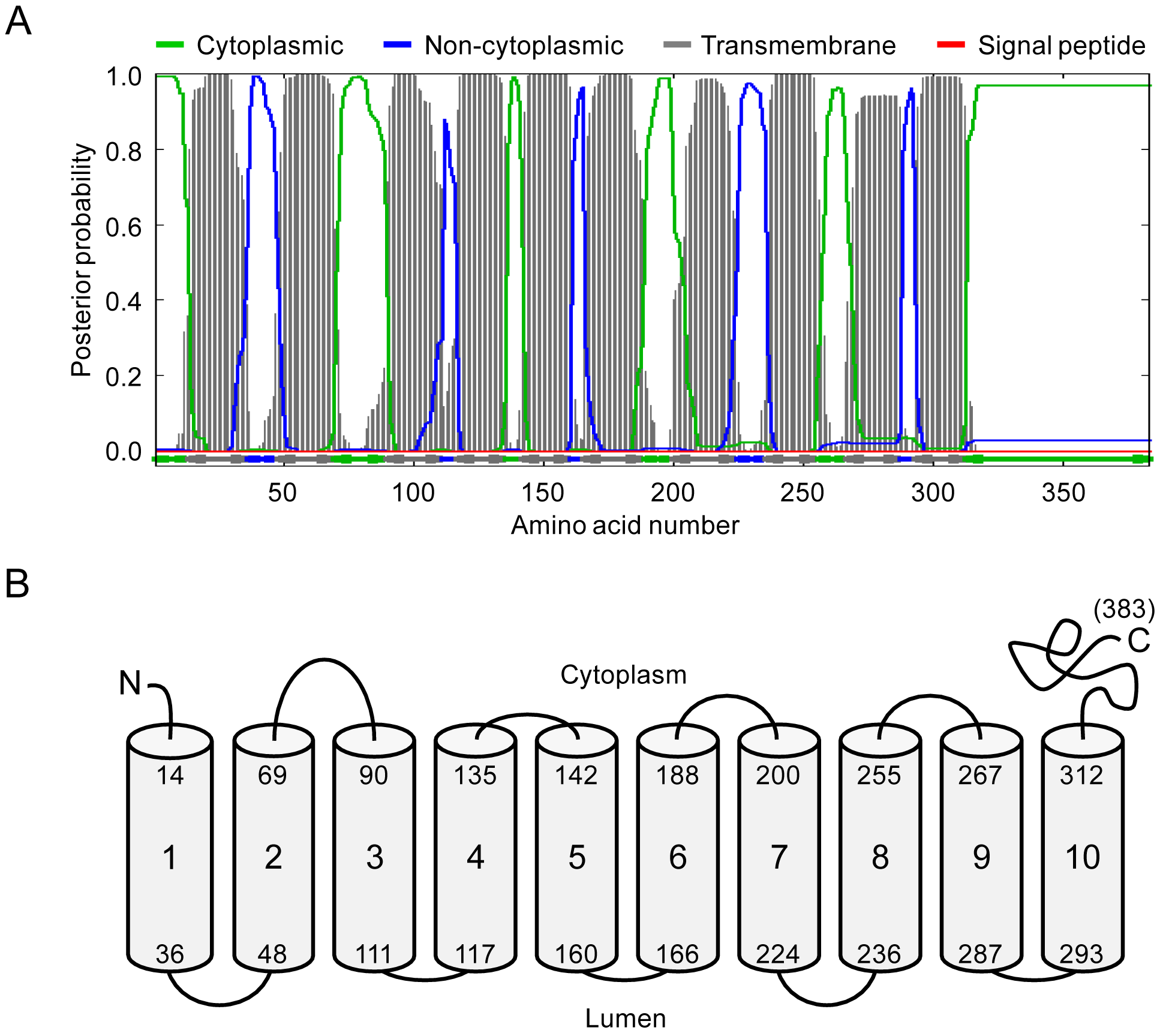

Supplement: Additional file 2: Figure S1 — This TIF document contains Additional file 2: Figure S1, which features the results of an in silico analysis of GFT membrane topology. Transmembrane domains were identified in the schistosome GFT protein using the Phobius transmembrane topology and signal peptide prediction server [107]. The Phobius output suggested 10 TMDs, a number that is consistent with GDP-L-fucose transporters of other organisms [27,28,30,70] (also see Figure 5) (A). A model based on this output was constructed, portraying the arrangement of the 10 TMDs (numbers indicating the amino acid boundaries of each TMD) as well as the most likely orientation for schistosome GMD within the Golgi membrane (B). [file 1756-3305-6-201-S2.tiff]

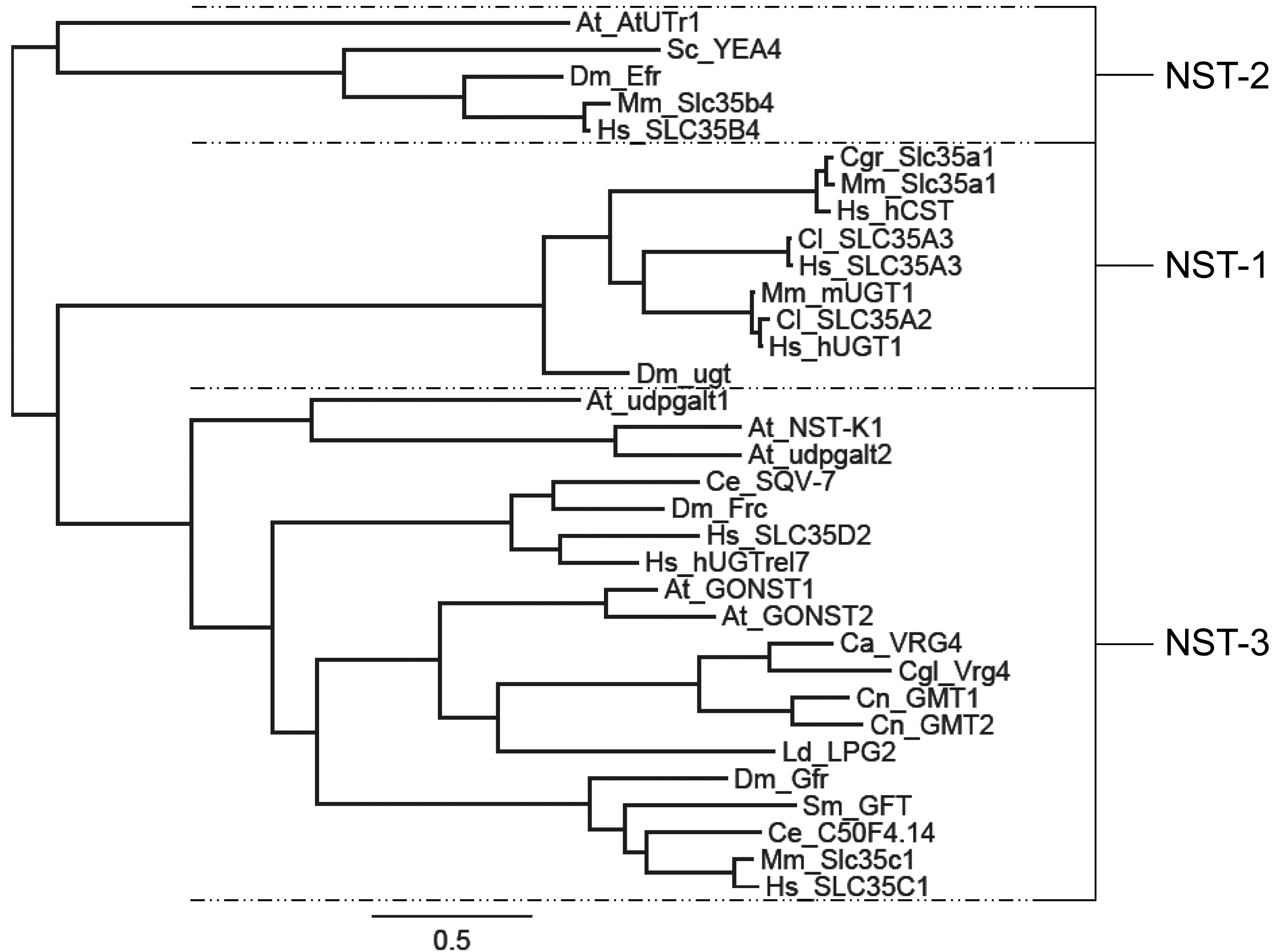

Supplement: Additional file 3: Figure S2 — This TIF document contains Additional file 3: Figure S2, which features a rooted phylogenetic tree of nucleotide-sugar transporters (see Figure 6 for detailed unrooted tree). The amino acid sequences of NSTs with previously characterized substrate specificities were obtained from RefSeq and GenBank databases at NCBI (accession numbers in Table 2). A tree was constructed using Bayesian methods implemented in MrBayes v3.12 with mixed amino acid evolutionary models. Monophyletic clades representing NST families 1–3 [108] are indicated, and genetic divergence (substitutions per site) is represented by the scale. The tree is rooted on NST family 2. [file 1756-3305-6-201-S3.tiff]

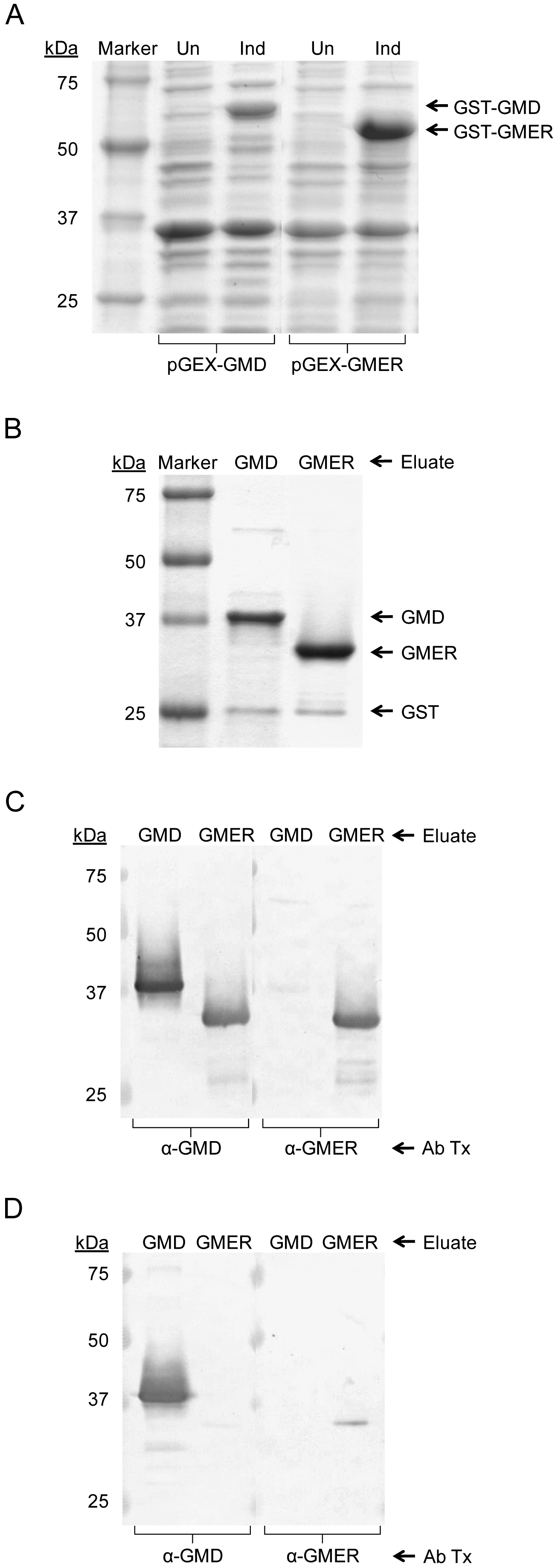

Supplement: Additional file 4: Figure S3 — This TIF document contains Additional file 4: Figure S3, which describes heterologous expression and isolation of recombinant schistosome GMD and GMER proteins and downstream affinity purification of GMD- and GMER-specific polyclonal chicken IgY. GST-GMD and -GMER fusion constructs were created in pGEX-6P-1 vector, and the encoded proteins were expressed in E. coli. Fusion protein expression in induced (Ind) and uninduced (Un) cultures was compared by SDS-PAGE fractionation and Coomassie staining of soluble cellular extracts (A). Fusion protein-containing extracts were passed through a GST-affinity column, and bound GMD and GMER were eluted by PreScission™ Protease-mediated cleavage of the GST fusions. Eluates were then analyzed by SDS-PAGE fractionation and Coomassie staining (B). Polyclonal chicken IgY antibodies were raised against recombinant GMD and GMER proteins, and the resultant antibodies were tested by immunoblotting the pure recombinant antigens (C). Due to crossreactivity among the antibodies and antigens (especially between anti-GMD IgY and recombinant GMER), antibodies were affinity-purified by membrane adsorption using bound GMD and GMER antigen. Following elution, antibody preparations were again tested against blots of pure antigen, demonstrating greatly reduced crossreactivity (D). [file 1756-3305-6-201-S4.tiff]
